# The herpetofauna of Timor-Leste: a first report

**DOI:** 10.3897/zookeys.109.1439

**Published:** 2011-06-20

**Authors:** Hinrich Kaiser, Venancio Lopes Carvalho, Jester Ceballos, Paul Freed, Scott Heacox, Barbara Lester, Stephen J. Richards, Colin R. Trainor, Caitlin Sanchez, Mark O’Shea

**Affiliations:** 1 Department of Biology, Victor Valley College, 18422 Bear Valley Road, Victorville, California 92395, USA; and The Foundation for Post-Conflict Development, 245 Park Avenue, 24th Floor, New York, New York 10167, USA; 2 Universidade National Timor-Lorosa’e, Faculdade de Ciencias da Educaçao, Departamentu da Biologia, Avenida Cidade de Lisboa, Liceu Dr. Francisco Machado, Dili, Timor-Leste; 3 14149 S. Butte Creek Road, Scotts Mills, Oregon 97375, USA; 4 Conservation International, PO Box 1024, Atherton, Queensland 4883, Australia; and Herpetology Department, South Australian Museum, North Terrace, Adelaide, South Australia 5000, Australia; 5 School of Environmental and Life Sciences, Charles Darwin University, Darwin, Northern Territory 0909, Australia; 6 West Midland Safari Park, Bewdley, Worcestershire DY12 1LF, United Kingdom; and Australian Venom Research Unit, Department of Pharmacology, University of Melbourne, Victoria 3010, Australia

**Keywords:** herpetofauna, biodiversity, Timor-Leste, Wallacea

## Abstract

Fieldwork conducted throughout Timor-Leste in September 2004 and July 2009 resulted in a collection or recording of 263 herpetological specimens (100 amphibians, 163 reptiles), comprising at least seven species of frogs and toads, 20 species of lizards, seven species of snakes, two species of turtles, and one species of crocodile. Among the amphibians, the most frequently encountered species were toads (*Duttaphrynus melanostictus*), rice paddy frogs (genus *Fejervarya*), and rhacophorid treefrogs (*Polypedates cf. leucomystax*). All three variants of rice paddy frogs encountered represent undescribed species similar to *Fejervarya verruculosa* from neighboring Wetar Island. Records of *Fejervarya cancrivora* and *Fejervarya limnocharis* for Timor Island are apparently errors based on misidentification. We obtained voucher specimens for a total of 147 lizards and voucher photographs only for four specimens of *Varanus timorensis*. Aside from geckos frequently associated with human habitations (e.g., *Gehyra mutilata*, *Gekko gecko*, *Hemidactylus frenatus*, *Hemidactylus platyurus*), we discovered an as yet undescribed species of bent-toed gecko, genus *Cyrtodactylus*, in the Same valley. Our specimens of *Hemidactylus platyurus* are the first record of this species from Timor-Leste. Commonly encountered skinks included four-fingered skinks (genus *Carlia*), wedge skinks (genus *Sphenomorphus*), and night skinks (genus *Eremiascincus*). Notable among the 15 snakes collected was the frequency of pitvipers (*Cryptelytrops insularis*), which amounted to over 25% of all snakes. Our specimen of the wolfsnake *Lycodon subcinctus* is the first record of this species for Timor-Leste. Based on these findings, it appears that the biodiversity of amphibians and reptiles in this remote corner of Wallacea is much greater than previously thought, particularly with respect to scincid lizards. The detail we provide in the species accounts is designed to allow the use of this report as a preliminary field guide to the amphibians and reptiles of Timor-Leste. However, survey work is ongoing.

## Introduction

Timor-Leste, also known as East Timor or Timor Lorosa’e, became the world’s newest country on 20 May 2002 when its independence was restored after 24 years of Indonesian occupation and three years of United Nations Transitional Administration. Prior to independence in 1975, the country had nominally been a Portuguese colony since 1702, after a continuous, though not unchallenged, Portuguese presence dating back to 1515. Its remote location in the farthest southeastern reaches of Wallacea, the biogeographic region delimited by Wallace’s Line to the west, Lydekker’s Line in the east, and the Timor Sea in the south (fide Bellwood 2007), posed logistical challenges to all but the most widely-traveled of biologists. The political uncertainty even during colonial times, particularly in the eastern part of the island, was compounded by guerrilla and Indonesian military activity over most of the past three decades and certainly proved a significant deterrent to biological research. The country’s rugged terrain and an infrastructure that still reels from the destruction wrought by the retreating occupation forces continue to make research a challenge. Thus, herpetological survey work in Timor-Leste is still in its infancy. As a consequence of the historical turmoil, there exist among the many dozens of specimens from Timor Island in major herpetological collections around the world very few from the eastern half of the island, the current territory of Timor-Leste. Here we present the results from Phase I of the first comprehensive survey of the amphibians and reptiles in independent Timor-Leste, along with an overview of the literature pertaining to the herpetofauna of the country and a glossary to improve the use of this paper by non-specialists.

### Geography and Geology.

**Figure 1.**
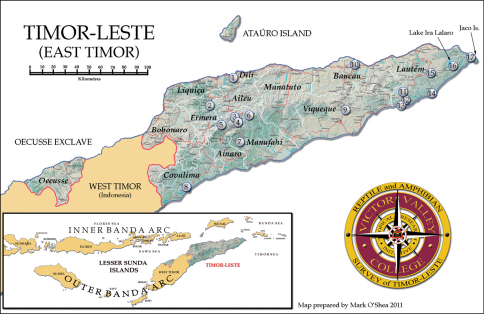
Map of districts, major towns, and collecting localities in Timor-Leste. Details for numbered localities are listed in Table 1. Map by Mark O’Shea.

Timor Island (Portuguese: Ilha de Timor; Bahasa Indonesia: Pulau Timor) is part of the Lesser Sunda Archipelago, an assemblage of islands composed of a northern, volcanogenic arc (the Inner Banda Arc, with main islands Bali, Lombok, Sumbawa, Flores, and Wetar) and a southern, orogenic arc (the Outer Banda Arc, with main islands Sumba, Roti, and Timor). These islands are located in an area of southeastern Wallacea (Fig. 1) where several biogeographic provinces converge. Timor Island is the largest landmass in the area (30,777 km2), and it emerged as a landmass during the Early Pliocene (ca. 5 mya) with a complex geologic history at the convergence of the Australian continental plate and the Eurasian continental landmass (ESDD 2003). The island is composed of a highly diverse stratigraphy, including several distinctive, monolithic limestone karst formations (e.g., the Paitxau Mountains). By its position as an oceanic landmass at the crossroads of the Southeast Asian and Australo-Papuan faunal realms, the Timor Island herpetofauna could be expected to comprise a mosaic of diversity created from these two main elements, with the addition of any endemic species.

The Democratic Republic of Timor-Leste occupies approximately the eastern half of Timor island (15,410 km2), with the inclusion of the Oecusse-Ambeno coastal exclave (Oecusse District; separated by an aerial distance of nearly 60 km west of the nearest point in Bobonaro District, Timor-Leste), Jaco Island at the easternmost tip of Timor (Lautém District; separated by a distance of less than 1 km from Tutuala Beach), and Ataúro Island (Dili District), 26 km north of the capital Dili. Whereas Ataúro (land area: 150 km2; Monk et al. 1997) is politically a part of Dili District in Timor-Leste, it is volcanogenic in origin (age: 3–3.5 mya), geographically part of the northern Banda Arc, and geologically distinct from Timor.

### Habitats.

Timor-Leste can be roughly divided into five major vegetation zones (Monk et al. 1997), including thorn forest (dry coastal areas, primarily along the north coast), dry deciduous forest (lower altitude habitats up to ca. 500 m), moist deciduous forest, semi-evergreen rainforest (especially on slopes), and evergreen rainforest (in the few pristine montane areas above 1000 m elevation). Trainor et al. (2007) provided a more detailed account of habitat types, which we follow here. Their classification includes tall evergreen forest (tree height up to 40 m), semi-deciduous and tropical dry forest types (tree height up to 20 m), a patchy tropical montane forest (elevations > 1000 m), beach forest and coastal scrub, savanna woodland, open eucalyptus forest, shaded coffee plantations (> 600 m), swamps and swamp forests, rice paddies, and village land. Habitats are generally characterized by sloping terrain (44% of the land in Timor-Leste has a slope of ≥ 40%), rendering them unsuitable for sustainable agriculture (UNDP 2010). Whereas Timor-Leste is typical of the tropics in possessing only a thin soil layer, there is little bare soil or grassland, and the island appears relatively well wooded.

There is little doubt that Timor-Leste was more forested before the arrival of the Portuguese colonists in the early 16th Century, but it is also apparent that some types of agriculture (such as the establishment of rice paddies) caused habitat modifications. However, as first the colonial power and then the Indonesian occupiers exploited tropical woods (notably sandalwood and teak), the effects of ongoing shifting subsistence agriculture became compounded. The reduction in the number of trees has by now dramatically increased the threat of erosion during the infrequent but often torrential rainfall, which may have serious consequences for road infrastructure. The threat of continued deforestation to support unsustainable agriculture techniques and the search for cooking fuel are real in Timor-Leste. These types of threats and the new threat of invasive species make sustainability efforts imperative.

### A brief history of herpetology in Timor-Leste.

Exploration of the Lesser Sunda Archipelago began in earnest shortly after the explorers Louis-Antoine de Bougainville (in 1768) and James Cook (in 1770) sailed past the island (see van Aken 2005 for a review). At this time, the well-established Dutch port of Kupang at the western end of Timor (“Coupang” or “Coepang” in writings of the time) appears to have been a favorite place for refitting on the globe-spanning voyages of the English commander Matthew Flinders (expedition dates: 1801–1803) and the French commanders Nicolas Baudin (1800–04), Louis-Claude de Freycinet (1817–20), Louis Isidore Duperrey (1822–25), and Dumont d’Urville (1826–29), all of whose vessels included naturalists and artists. It is remarkable that any results at all were eventually reported from these voyages, since many of the travelers died from diseases shortly after reaching Southeast Asia (among them the very active Dutch collectors Heinrich Christian Macklot, Heinrich Boie, and Heinrich Kuhl).

The years from 1800–1830 may therefore be considered the first wave of scientific exploration to the shores of Timor. During this initial wave, the most significant collections in general, and on Timor in particular, were made by the French (e.g., François Péron and Charles-Alexandre Lesueur traveling with Baudin, Jean René Constant Quoy and Joseph Paul Gaimard traveling first with Duperrey and then d’Urville). Whereas Baudin landed only in Kupang (in 1803), Duperrey visited both Kupang and Dili (“Diely” or “Dielly” in writings of the time) in 1818. Shortly thereafter (1828), the Dutch vessel *Triton* landed in Kupang with naturalists Macklot, Boie, and Salomon Müller, who spent several years in the Lesser Sundas.

Even though in the late 1850s some amphibians and reptiles from Timor were sent to the Dutch Rijksmuseum van Natuurlijke Historie (RMNH) by Pieter Bleeker, a medical officer stationed in the Dutch East Indies, the second wave of exploration began in earnest only with the explorations of the Swiss zoologists Paul Benedict Sarasin and Fritz Sarasin, who traveled to the Lesser Sundas in 1893–96 and 1902–03, and continued with the *Siboga* Expedition (1898–1900) under the leadership of Max Weber (RMNH). This period also saw the publication of the only substantial Portuguese herpetological reports on Timor (Bethencourt Ferreira 1897, 1898), the collections of Thomas Barbour (1906–07; Museum of Comparative Zoology, Cambridge, Massachusetts, USA), the extended museum-based reports by Nelly de Rooij (de Rooij 1915, 1917), as well as the collections of Felix Kopstein (1922–24; RMNH), Malcolm Smith (1924, British Museum of Natural History, London, United Kingdom), Emmett Reid Dunn (1926; American Museum of Natural History, New York, USA), and Robert Mertens (1927; Forschungsinstitut und Naturmuseum Senckenberg, Frankfurt am Main, Germany). After this second wave had passed, a period of political instability commenced that included the warfare of the 1940s, the period of decolonization during the 1960s and 1970s, and the subsequent annexation by Indonesia, which halted research on Timor almost entirely (with the exception of a collection made in West Timor by researchers from the Western Australian Museum in the early 1990s) until Timor-Leste reached a modicum of political stability within a few years of the 1999 UN-sponsored referendum on independence.

Timor Island and the southeastern corner of Wallacea are a particularly interesting locale from a biogeographic point of view, but the relative influence of Southeast Asian and Australo-Papuan elements on the herpetofauna has not been studied. Positioned on the rugged eastern half of the largest landmass in the area (Timor is the 44th largest island in the world and the 7th largest between Southeast Asia and New Guinea), Timor-Leste is a likely source of significant endemism and well-positioned to begin an assessment of the biogeographic provenance of its herpetofauna. In recognition of this need, and given a near absence of herpetofaunal information for the country, we resolved to begin a comprehensive survey. This report summarizes the results of a survey in 2004 (by SJR) and Phase I of a series of ongoing surveys conducted by the other authors (except CRT and SJR) in July 2009.

## Materials and methods

We collected amphibians and reptiles in Timor-Leste from 2–12 September 2004 (SJR only) and from 15–30 July 2009 at 18 localities throughout the country (Table 1; Fig. 1). Even though the country currently has not acceded to the CITES treaty, we decided *a priori* to document the presence of known monitor lizards, pythons, and snake-necked turtles primarily via photographic vouchers or from road-kills because of their CITES Appendix II status, unless extenuating circumstances required adjustment of this procedure. Were the species status of CITES-listed taxa in question, we would only collect a minimal number of specimens and/or their tissues to ascertain their taxonomic status. All other amphibians and reptiles were sampled and a sub-set of specimens encountered was preserved as vouchers. During our survey we encountered some animals that were not readily identified to species despite careful comparisons with type material and examination of pertinent taxonomic literature. In cases where there seemed to be superficial resemblance to described taxa but where comparisons were complicated by the unavailability or age of comparative material, by incongruities with our own field experience, or by pending taxonomic revisions, we flagged the situation by inserting the clause “cf.” (Latin: *confer* = compare) into the species name (e.g., *Polypedates cf. leucomystax*). Specimens not matching any known species and presumed to be species new to science (candidate species, fide Padial et al. 2010) are first listed using the abbreviation “sp.” followed by an integer and a candidate designation with a specimen number. For example, an undescribed species of *Cyrtodactylus* would be listed as *Cyrtodactylus* sp. 1 [Ca CMD 383], where CMD 383 is the field number of a key specimen. Subsequent mentions of this candidate species in the text will use only the abbreviation “sp.” with its assigned integer.

**Table 1.** Localities surveyed in Timor-Leste during September 2004 and July 2009, including GPS coordinates.

*Locality Number*	*District*	*Locality*	*Elevation (m)*	*GPS1*
1	Dili	area of Dili town and surrounds	1–20	S 08°33', E 125°32'
2	Ermera	Eraulo village (Sta. Bakhita Mission)	1200	S 08°47', E 125°27'
3	Ainaro	Eralisau village, near Maubisse	901–1526	S 08°50', E 125°35'
4	Ainaro	area of Maubisse town and surrounds	1484	S 08°51', E 125°36'
5	Ainaro	slopes of Mount Ramelau	831–2960	S 08°52', E 125°30'
6	Manufahi	Turiscailau village, near Maubisse	1225	S 08°50', E 125°44'
7	Manufahi	area of Same town and surrounds	513–554	S 09°00', E 125°39'
8	Covalima	area of Suai town and surrounds	13	S 09°19', E 125°15'
9	Viqueque	Timor Village Hotel and surrounds	285	S 08°47', E 126°23'
10	Baucau	area of Baucau town and surrounds	5–350	S 08°28', E 126°28'
11	Lautem	bat cave, Iliomar subdistrict	285	S 08°39', E 126°50'
12	Lautem	area of Iliomar town and surrounds	315	S 08°43', E 126°50'
13	Lautem	40 min S Iliomar by road	not determined	S 08°45', E 126°49'
14	Lautem	area of Lore 1 village and surrounds	3	S 08°41', E 126°59'
15	Lautem	area ofLospalos town and surrounds	340	S 08°31', E 127°00'
16	Lautem	5 km S Mehara village	125	S 08°27', E 127°10'
17	Lautem	Tutuala beach (Pantai Walu)	4	S 08°25', E 127°17'

^1^ GPS coordinates are approximate to define the area in which the survey work was carried out. Exact localities are not provided to protect some of the unique and fragile habitats in Timor-Leste.

### Localities.

Whereas our surveys were conducted entirely within the country of Timor-Leste, historical collections were concentrated in the western part of the island near the historic port of Kupang. To avoid confusion, we refer to Timor when considering the entire island, but we may differentiate West Timor, defined as the western portion of Timor and politically part of the Indonesian province East Nusa Tenggara (Bahasa Indonesia: Nusa Tenggara Timur), from Timor-Leste (the sovereign nation). Timor and Timor-Leste should not be confused, as has sometimes happened, with Timor Laut, a name used for the Tanimbar Islands of Indonesia’s Maluku province to the northeast.

**Figure 2.**
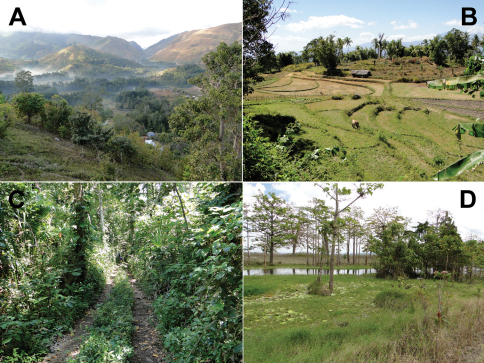
Examples of sampled habitats in Timor-Leste. **A** Highland habitat in the area of Maubisse, Ainaro District (altitude at the level of the buildings ca. 1400 m). This habitat has experienced considerable deforestation, as evidenced by the presence of small forest patches in the low-lying areas and the absence of trees on the higher slopes of Mt. Ramelau, in this view. This deforestation apparently began only in the early 1980s (Trainor et al. 2007). The area supports a very active coffee industry. The patchwork of forest, river valleys, coffee plantations, and deforested slopes creates a mosaic environment that most likely will cater exclusively to habitat generalist species **B** Tiered rice paddies south of Baucau, Baucau District. The area surrounding Baucau is a classic rice-growing region at low to moderate altitudes (sea level–500 m). In this type of habitat we commonly encountered rice paddy frogs as well as their snake predators, and some of the perianthropic geckos. Many of these terraced rice paddies have been operational for hundreds of years, and they are a disturbed habitat exposed to strict human-initiated seasonality (a wet growing season with artificial vegetation and irrigation, a dry fallow season with dry soil and absence of any vegetation) **C** Road leading through a lowland tropical evergreen forest (Trainor et al. 2007) on the southeastern coast near Loré, Lautém District (altitude near sea level). This area also supports coastal dry forest, tidal forests including mangroves, and coastal grasslands. The distance of this area from major population centers and its relative inaccessibility may be the primary reasons for the presence of such a diverse set of intact habitats **D** The Lake Ira Lalaro floodplain and surrounding area. Whereas the foreground of this image shows the marshy edges of the Irasequiro River, the background shows the treeless expanse of the lake’s floodplain. Because this area is a highly seasonal water source and prone to flooding, it has experienced very little development. Photos by Hinrich Kaiser.

We conducted our surveys at localities (Table 1) that cover the major habitat types and the diverse geography of the country (Fig. 2). Choice of localities was nearly always influenced by the presence of supporting infrastructure (i.e., vehicle access, accommodation, electricity) to facilitate collecting and processing of specimens. Since Timor-Leste regained independence in 2002 (after a 7-day period in late 1975 just prior to Indonesian annexation), efforts have been underway to reconstruct and solidify infrastructure, such as roads, electricity, and telephone networks, but much of the existing infrastructure remains in poor condition. This is especially the case the further one travels from the capital, Dili.

### Collecting.

Survey protocols involved active searches along roadsides, forest paths, and in riverbeds, supplemented by collection of road-kills and specimens encountered fortuitously while driving. Searches were conducted during the day and at night. During the day we scanned the ground, tree trunks, and foliage, capturing frogs, lizards, and non-venomous snakes by hand. We used neoprene-padded M1 snake tongs and a Pro Bagger (Midwest Tongs, Independence, Missouri, USA) to handle, capture, and transport venomous snakes. Lizards high in trees were stunned with plastic plugs shot from 6-foot (183 cm) blowguns (Blowguns Northwest, Richland, Washington, USA). We also removed bark from rotting logs and turned them over with stump rippers (Midwest Tongs), carefully dismantled rock piles and similar potential refugia by hand, shone flashlights into nooks and crannies, and raked through leaf litter. All logs and rocks were replaced as closely as possible to their original position. By night we used hand-held flashlights to search the ground, along the edges of watercourses and swamps, and in the vegetation. As our collecting activities became known to the local population, we were occasionally presented with specimens deemed worthy of our attention (one lizard, two snakes, two turtles). These specimens were offered and accepted with no request for remuneration. At the time of capture, we recorded date, time, altitude, circumstances of capture, and GPS coordinates for each locality using a Garmin Oregon 400t (Garmin International Inc., Olathe, Kansas, USA). In order to convey the relative frequency of observations (Table 2), we classified species encounters as abundant (many encounters throughout a locality), common (usually present at a locality), infrequent (few individuals seen, or unpredictable), rare (seen once only), or indeterminate (in cases where our field experience and knowledge of the local population was insufficient to permit classification).

### Processing.

During all surveys, at least one individual of each captured species at each locality was photographed. Whenever possible, we also photographed the specimen *in situ* before capture. After capture, specimens were set up on a set, in a 90-cm Cubelite (Lastolite Ltd., Coalville, Leicestershire, United Kingdom) using habitat materials, and photographed in several positions to capture morphological detail in life, and to ensure a record of all possible color patterns in life. Due to the vagaries of specimen preparation in the field, we were unable to connect all photographic vouchers shown in the figures to specimen numbers. Where possible, specimen numbers are included in the figure captions.

Specimens were euthanized by intracardiac injection with a 5% procaine solution (Altig 1980) or by immersion in dilute chlorobutanol (frogs only), following standard animal care protocols (e.g., ASIH 2004; Animals for Research Act Canada, RRO 1990, Regulation 24). Liver tissue samples were removed from most voucher specimens through small lateral incisions and preserved in 1.5-ml plastic screw-cap centrifuge tubes containing 95% non-denatured ethanol (EtOH). Ancillary collections of external and internal parasites (mites, ticks, tapeworms, nematodes) were made opportunistically when such organisms were discovered during specimen processing. In instances where captive individuals defecated into their plastic bag, fecal samples of the fresh material were preserved in 2.5% aqueous (w/v) potassium dichromate (K2Cr2O7) according to the method described by Duszynski et al. (1982). Immediately after euthanasia, snout-vent length (SVL) and total length (TL) were measured for all reptiles to ensure that specimens could be laid out straight and measured accurately. We also took some scale counts to aid in the identification of species. Specimens were sexed before preservation when possible by everting hemipenes or by checking for the presence of ovaries or yolked eggs. For lizards, data on maturity and reproductive condition were made after preservation and will be reported elsewhere (Goldberg et al., in prep.).

All specimens were fixed in 10% formalin. Amphibians were placed directly into plastic tubs and carefully positioned, whereas reptiles were first injected with 10% formalin and then positioned. Specimens were then covered with formalin-saturated paper towels before the tubs were sealed and the specimens left to set. After fixing, field-numbered series of potentially problematic taxa (e.g., gekkonids, scincids, anurans) were photographed in dorsal and ventral view for later comparative study. Voucher specimens of amphibians and reptiles (Appendix I) have been deposited in the Division of Amphibians and Reptiles, National Museum of Natural History, Smithsonian Institution, Washington DC, USA (USNM) and the South Australian Museum, Adelaide, Australia (SAMA). At the time of publication, some specimens are listed for the USNM using CMD field numbers, as these have not yet been accessioned. However, these specimens are scheduled to become permanently integrated into the USNM collection by August 2011, at which time they will be accessible for research. Furthermore, searches of the USNM specimen database using these field numbers will reliably yield the specimens designated by these numbers herein.

### Supplement.

In addition to the information provided in this paper, a brief glossary of relevant scientific terms is provided on the *Zookeys* website to augment public understanding of the information presented herein, and to allow local authorities the use of this paper as an instrument for recognizing the herpetofauna of Timor-Leste.

**Table 2.** Checklist of the species of amphibians and reptiles currently verified for Timor Island. The list of synonyms comprises those scientific names that have been applied to Timor populations and is not an exhaustive list of synonyms for the species concerned. The most commonly used authorities are abbreviated as Ba = Barbour (1912), BF97 = Bethencourt Ferreira (1897), BF98 = Bethencourt Ferreira (1898), Bl = Bleeker (1860), dH = de Haas (1950), dR15 = de Rooij (1915), dR17 = de Rooij (1917), F = Forcart (1953), IC = Iskandar and Coljin (2001), S = Smith (1927), vK = van Kampen (1923), vL = van Lidth de Jeude (1895), Victor Valley College survey = VVC. We consider as verified the occurrence of species either by our collection or by the presence of voucher specimens in museum collections that we have seen. Species in boldface print are confirmed for Timor-Leste. Altitudes listed are for specimens we collected. Habitat types follow Trainor et al. (2007) and are abbreviated as TEF = tall evergreen forest, TDF = tropical dry forest, TMF = tropical montane forest above 1000 m altitude, BFCS = beach forest and coastal scrub, SW = savannah woodland, EF = eucalyptus forest, CP = coffee plantations, SW = swamp and swamp forest, RP = rice paddies, and VIL = village land. Frequencies listed are of those species we found in Timor-Leste, defined as abundant (many encounters at specific locality), common (usually present at a specific locality), infrequent (few individuals seen, or unpredictable), rare (seen once), or indeterminate (in cases where our field experience and the knowledge of the local population were insufficient to permit classification).

*Current Name*	*Synonyms (Authority)*	*Altitude (m)*	*Habitat(s)*	*Frequency*
Frogs and Toads				
Family Bufonidae				
*Duttaphrynus melanostictus*	*Duttaphrynus melanostictus* (VVC)	0–600	TDF, BFCS, SW, CP, RP, VL	abundant
	*Bufo melanostictus* (Trainor et al. 2009)			
Family Dicroglossidae				
*Fejervarya* spp.	*Rana tigrina* (Bl)	0–1200	TEF, TMF, CP, SW, RP, VL	abundant
	*Rana tigerina var. verruculosa* (Roux 1911)			
	*Rana tigerina* (Ba)			
	*Rana verruculosa*1 (S, vK, Menzies 1987)			
	*Rana cancrivora* (S, vK)			
*Limnonectes timorensis*	*Limnonectes timorensis* (VVC)	> 1000	TMF	infrequent
	*Rana timorensis* (S)			
	*Hylarana elberti* (F, Menzies 1987)			
Family Hylidae				
*Litoria everetti*	*Litoria everetti* (VVC)	> 1000	TMF	infrequent
	*Hyla everetti* (Ba, S, vK),			
Family Rhacophoridae				
*Polypedates cf. leucomystax*	*Polypedates cf. leucomystax* (VVC)	0–1400	TEF, TDF, TMF, SW, EF, CP, SW, VL	common
	*Polypedates leucomystax* (Ba, Bl, vK)			
	*Rhacophorus leucomystax* (vK)			
	*Rhacophorus leucomystax* var. *sexvirgata* (vK)			
Lizards				
Family Agamidae				
*Draco timoriensis*	*Draco timoriensis* (VVC, Ba, Bl, dR15)	0–300	TEF, TDF, BFCS, VL	
	*Draco timorensis* (BF98, vL, Manaças 1972),			
	*Draco viridis* var. *timoriensis* (Schlegel 1837–44)			
	*Draco haematopogon* (Bl)			
	*Draco lineatus* (Bl)			
	*Draco volans* (dR15)			
	*Draco walkeri* (Ba, dR15)			
Family Gekkonidae				
*Cyrtodactylus* sp.	*Cyrtodactylus* sp. (VVC)	600	TEF, CP	rare
	*Goniodactylus Timorensis*2(Boulenger 1885)			
	*Gymnodactylus (?) marmoratus* (S)			
*Gehyra cf. mutilata*	*Gehyra cf. mutilata* (VVC)	Lowlands	TDF, BFCS, SW, EF, RP, VL	infrequent
	*Gehyra mutilata* (dR15)			
	*Peropus mutilatus* (S)			
	*Hemidactylus platurus*3 (Bl)			
*Gekko gecko*	*Gekko gecko* (Ba, VVC)	0–300	TEF, TDF, BFCS, SW, EF, CP, VL	common
	*Gekko verticillatus* (BF98, dR15, vL, Manaças 1972)			
	*Platydactylus guttatus* (Bl)			
*Hemidactylus frenatus*	*Hemidactylus frenatus* (BF98, Ba, Bl, dR15, VVC)	0–300	TDF, BFCS, SW, EF, CP, RP, VL	abundant
*Hemidactylus platyurus*	*Cosymbotus platyurus* (VVC)	0–300	TDF, BFCS, SW, VL	infrequent
Family Scincidae				
*Carlia* spp.	*Carlia* spp. (VVC)	0–1500	TDF, TMF, BFCS, VL	common
*Carlia peronii*	*Carlia peronii* (Zug 2010)			
	*Heteropus peronii* (Duméril and Bibron 1839)			
*Carlia spinauris*	*Carlia spinauris* (Zug 2010)			
	*Lygosoma* (*Leiolopisma*) *spinauris* (S)			
*Cryptoblepharus leschenault*	*Cryptoblepharus leschenault* (VVC)	Lowlands	TDF, BFCS	infrequent
	*Ablepharus boutonii leschenault* (F, Mertens 1930)			
*Eremiascincus* spp.	*Eremiascincus* spp. (VVC)	0–2100	TDF, TMF, BFCS	common
				
*Eremiascincus antoniorum*	*Glaphyromorphus antoniorum* (Greer 1990)			
	*Lygosoma* (*Omolepida*) *antoniorum* (S)			
*Eremiascincus timorensis*	*Glaphyromorphus timorensis* (Greer 1990)			
*Eutropis cf. multifasciata*	*Eutropis cf. multifasciata* (VVC)	0–1200	TDF, SW, EF, CP, VL	common
	*Mabuya multifasciata* (dR15, S)			
	*Euprepes Sebae* (Bl)			
*Lamprolepis cf. smaragdina*	*Lamprolepis cf. smaragdina* (VVC)	0–300	TDF, BFCS, SW, EF, VL	common
	*Lygosoma smaragdinum* (Bl, dR15, vL)			
	*Dasia smaragdinum* (Ba)			
*Sphenomorphus* spp.	*Sphenomorphus* spp. (VVC)	0–1500	TEF, TDF, TMF, BFCS	infrequent
Family Varanidae				
*Varanus timorensis*	*Varanus timorensis* (BF98, dR15, S, vL, Boulenger 1885, VVC)	Lowlands	BFCS, SW, RP, VL	infrequent
	*Varanus timoriensis* (Ba, Bl)			
Snakes				
Family Colubridae				
*Coelognathus subradiatus*	*Coelognathus subradiatus* (VVC)	0–400	TDF, VL	infrequent
	*Coluber melanurus*4 (dR17)			
	*Coluber melanurus* var. *timoriensis* (BF97, BF98, dR17)			
	*Coluber subradiatus* (dR17, S)			
	*Compsosoma melanurus*4 (Bl)			
	*Compsosoma subradiatum* (Bl)			
	*Elaphe melanura* (Ba)			
	*Elaphe subradiata* (dH, Schulz 1996)			
	*Elaphe timoriensis* (Ba)			
	*Elaphis subradiatus* (vL)			
*Dendrelaphis inornatus timorensis*	*Ahaetulla picta inornata*5(dH)	0–300	TDF, BFCS	infrequent
	*Dendrelaphis inornatus timorensis* (F), VVC)			
	*Dendrophis picta* (Bl)			
	*Dendrophis pictus* (Ba, BF98, dR17, vL)			
	*Dendrophis pictus timorensis* (S)			
*Lycodon capucinus*	*Lycodon aulicum* (Bl)	500	TDF, VL	indeterminate
	*Lycodon aulicus* (Ba, dR17, S)			
	*Lycodon aulicus capucinus* (dH)			
	*Lycodon aulicus* var. D (BF98, Boulenger 1893)			
	*Lycodon capucinus* (VVC)			
*Lycodon subcinctus*	*Lycodon subcinctus* (VVC)	500	VL	indeterminate
Family Homalopsidae				
*Cerberus rynchops*	*Cerberus rynchops* (BF98, dR17, VVC)	Lowlands	RP	infrequent
	*Cerberus rynchops rynchops* (dH)			
	*Cerberus boaeformis* (Bl)			
	*Hurria rhynchops* (Ba)			
Family Typhlopidae				
*Ramphotyphlops braminus*	*Ophthalmidion crassum*6(Bl)	0–300	TDF, VL	infrequent
	*Ramphotyphlops braminus* (VVC)			
	*Typhlops braminus* (Ba, dH, dR17, vL)			
Family Viperidae				
*Cryptelytrops insularis*	*Cryptelytrops insularis* (VVC)	0–500	TDF, BFCS, EF, RP, VL	infrequent
	*Bothrops erythrurus* (vL)			
	*Bothrops viridis* (Bl)			
	*Lachesis gramineus* (BF98, dR17, Manacas 1972)			
	*Trimeresurus albolabris* (dH)			
	*Trimeresurus gramineus* (Ba)			
Turtles				
Family Chelidae				
*Chelodina timorensis*	*Chelodina timorensis* (McCord et al. 2007)	300	Lake Ira Lalaro	rare
	*Chelodina mccordi timorlestensis* (Kuchling et al. 2007)			
	*Chelodina novae-guineae* (Ba)			
Family Geoemydidae				
*Mauremys reevesii*	*Mauremys reevesii* (Kaiser et al. 2010)	0-300	VL	rare
CroCodiles				
				
Family Crocodylidae				
*Crocodylus porosus*	*Crocodilus biporcatus* (Bl)	Lowlands	SW	common
	*Crocodilus porosus* (BF98)			
	*Crocodylus porosus* (Ba, dR17)			

^1^ Menzies (1987) recorded vocalizations of rice paddy frogs, which he called a short call (Menzies 1987: Fig. 16b) and a long call (Menzies 1987: Fig. 16c). We have heard these types of calls and traced them to males of what we believe to be two distinct species of *Fejervarya*.

^2^ Gray’s (1845) and Boulenger’s (1885) Catalogues list this species in the synonymy of what is now known as *Cnemaspis boiei*, a gecko from India. Gray’s description of the specimen from Timor reads thus: “In spirits. Wants tail. Timor ? Presented by T. Bell, Esq.” The fact that this tailless specimen was from a different collection than that received by Duméril and Bibron in Paris, which itself consists of only a single bent-toed gecko specimen from Timor, raises the possibility that a species of *Cyrtodactylus* or *Cnemaspis* other than the one described by Duméril and Bibron (1837) was discovered by Thomas Bell among the material collected on the voyages of HMS Beagle. Gray (1845) writes that the specimens procured by Darwin and Captain Fitzroy were presented to the British Museum of Natural History by Bell after describing them in his volume titled *Zoology of the HMS Beagle*. The specimen listed by Gray (1845) is presumed lost (C. McCarthy, in litt.).

^3^ Bleeker (1859) provided a detailed listing of a form he named *Hemidactylus platurus*. This form is related to *Gehyra mutilata* based on the original description, and it is therefore not to be confused with *HHemidactylus platyurus*. However, since no type specimen was designated, the name *Hemidactylus platurus* Bleeker (1859) is a nomen nudum.

^4^ The names *Coluber melanurus*, *Compsosoma melanurus*, and *Elaphe melanura* are currently in the synonymy of *Coelognathus flavolineatus*. Absent any specimens of *Coelognathus flavolineatus* from Timor and given its known range, we consider all reports of this species on Timor in error and refer them to *Coelognathus timoriensis*.

^5^ According to How et al. (1996), *Ahaetulla picta inornata* is a synonym of *Dendrelaphis inornatus timorensis*.

^6^ Bleeker (1860) listed this name in error for what is undoubtedly a reference to *Ramphotyphlops braminus*. The name *Ophthalmidion crassum* is actually a synonym of the South American blindsnake *Typhlops reticulatus*.

## Species accounts

In the following accounts, we provide the most current accepted scientific name for each species based on literature available as of 1 September 2010. Each species name is given with its author to make its taxonomy unequivocal. A more complete checklist of species known from Timor and Timor-Leste with synonyms used to document their presence is provided in Table 2. Beneath the species name we list common names in English (E), taken from or modeled according to common usage by professional herpetologists, and in Tetun (T) and any local language for which a name is commonly in use. Tetun names with an asterisk (*T) are newly coined and designed to approximate the English name. Words in Tetun are spelled based on Hull (2001), with localities spelled according to common usage and in recognition of historic names (C. Williams-van Klinken, in litt. 20 July 2010). Our accounts include brief descriptive statements to aid field identification, taxonomic comments, information regarding the natural history of species, and any additional comments that may assist the reader’s understanding of the species’ occurrence in Timor-Leste. The list of specimens examined is provided in Appendix I in the same order as species are listed in the main text.

### Frogs and Toads (Order Anura)

#### Family Bufonidae - True Toads

##### Duttaphrynus melanostictus

(Schneider, 1799)

http://species-id.net/wiki/Duttaphrynus_melanostictus

Fig. 3

###### Common names.

(E) Black-spined Toad, Common Asian Toad, Common Sunda Toad. (T) Manduku Interfet (manduku = frog, INTERFET = International Force for East Timor; see below).

###### Identification.

**Figure 3.**
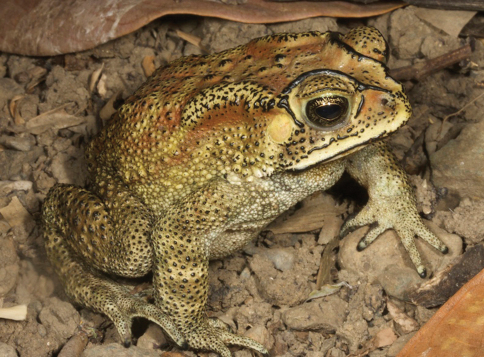
*Duttaphrynus melanostictus*. Yellow morph from Same, Manufahi District (SVL 53 mm). Photo by Mark O’Shea.

This toad can be recognized by its stout body, dry and warty skin, and by the distinct pattern of bony ridges (cranial crests) on the head. The shape and color of these ridges are characters useful for distinguishing among different toad species. In *Duttaphrynus melanostictus* they are of varying thickness and include a canthal ridge, supralabial ridge, and a series of ridges framing the eye (preorbital, supraorbital, postorbital, and orbito-tympanic ridges; Fig. 3). The tops of these ridges are usually black. A second important and useful characteristic to differentiate between toad species is the size and shape of the large parotoid gland (sometimes also described as a “poison” gland) on either side of the head. In *Duttaphrynus melanostictus* this gland is elongate and about 2½–3 times the size of the eye (Fig. 3). This species of toad also usually has several protruding wart-like skin glands on its back behind its head in addition to many smaller glands all over its body, most of which are tipped with black. Our familiarity with the species from elsewhere in the region allows us to confirm its identity.

###### Collection and natural history.

Hiding under a diverse array of objects by day and active by night, this toad is a relatively recent invader of Timor-Leste (see Trainor 2009), yet it was one of the most commonly encountered amphibians where it occurred. The distribution of *Duttaphrynus melanostictus* in Timor-Leste currently excludes areas of high elevation (above ca. 1200 m) as well as the region east of a line connecting Manatuto and Viqueque (Trainor 2009). We collected five voucher specimens at night (e.g., on the path leading to the Trilolo River north of Same, Manufahi District, altitude 553 m) and noted the presence of this species in disturbed habitats (e.g., towns, roadsides), cultivated habitats (e.g., coffee plantations) and some fairly pristine habitats (e.g., coastal scrub). Individuals ranged from tadpoles and juveniles (though not recent metamorphs) to adults and they exhibited varying shades of dull yellow to brown coloration. This is the only species of true toad reported from Timor-Leste so far, but we have been unable to verify the presence of specimens from Timor in herpetological collections. Therefore, our records appear to be the first vouchered confirmation of this species for Timor-Leste and Timor.

###### Toad introductions.

Reports by Australian peacekeepers of the cane toad, *Rhinella marina*, in Timor-Leste are an error arising from the soldiers’ familiarity with *Rhinella marina*, the only bufonid introduced to Australia and New Guinea, and their lack of familiarity with the Asian *Duttaphrynus melanostictus*. It is interesting in this regard that this species has taken on the Tetun name of the International Force for East Timor (INTERFET), the transitional peacekeeping force that arrived to stabilize the country after the departure of the Indonesian occupation force. INTERFET was composed primarily of Australian peacekeepers and the local belief, based on the erroneous identification of *Duttaphrynus melanostictus* by these personnel, is that INTERFET is responsible for the presence of this toad in Timor-Leste. The distribution of *Duttaphrynus melanostictus* includes several other Lesser Sunda Islands (e.g., Bali, Lombok), and it may be impossible to ascertain from where and when the initial wave of toad invasion originated.

The introduction of toads to non-native environments has frequently resulted in ecological disasters. The most notorious example of this has been the cane toad (*Rhinella marina*), whose spread by humans has become a problem with nearly global implications (e.g., Covacevich and Archer 1975; Lever 2001; Phillips et al. 2007). The species has been carried from its native northern South American habitat to locations as widespread as the Greater and Lesser Antilles, Florida, Hawaii, the Fiji Islands, the Philippines, Taiwan, the Ryukyu Island Archipelago of Japan, several Pacific islands, New Guinea, and, famously, Australia (see Zug and Zug 1979). It appears that the spread of *Duttaphrynus melanostictus* may rank a close second in terms of its geographic reach (from the Indian subcontinent throughout mainland and insular Southeast Asia), though perhaps not in terms of its ecological significance (e.g., Inger and Voris 2001). However, toads are voracious opportunistic predators whose impact on a newly colonized ecosystem may take years to assess. Reported impacts include alteration of the food chain, detrimental effects on lizard population recruitment, extirpation of leaf litter amphibians and their tadpoles, reduction of amphibiophagous reptile and mammal densities, and even poisoning of human or canid predators (e.g., Trainor 2009). We have recommended to government agencies that the advance of *Duttaphrynus melanostictus* in Timor-Leste requires close monitoring and a popular campaign to avoid human injury. A second toad invasion appears to be underway concurrently by *Ingerophrynus biporcatus* (formerly *Bufo biporcatus*) on Roti Island, an island neighboring Timor (Trainor 2009).

A simple distinction between *Duttaphrynus melanostictus* and *Rhinella marina* can be made by looking at the morphology of features described above. Whereas *Duttaphrynus melanostictus* has an elongated parotoid gland that is about three times the size of the eye (Fig. 3), the gland of *Rhinella marina* is considerably larger (nearly five times the size of the eye) and shaped like an irregular rectangle with rounded corners. The cranial crests of *Duttaphrynus melanostictus* are relatively thin and topped with black, whereas those of *Rhinella marina* are rather stout, surround the eye and are colored as the rest of the head.

###### Taxonomic comment.

Prior to the revision of amphibian taxonomy by Frost et al. (2006), this species was known as *Bufo melanostictus*.

#### Family Dicroglossidae - Fork-tongued Frogs

##### Genus Fejervarya

http://species-id.net/wiki/Fejervarya

Fig. 4

###### Common names.

(E) Rice Paddy Frogs. (T) Manduku natar (manduku = frog, natar = rice paddy).

###### Identification.

**Figure 4.**
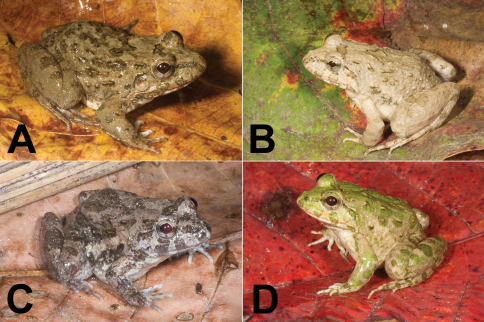
Rice paddy frogs, genus *Fejervarya*. **A** *Fejervarya* sp. 1 from the Baucau area (SVL 58 mm) **B** *Fejervarya* sp. 2 from the Viqueque area (SVL 46 mm) **C** *Fejervarya* sp. 3 from the Viqueque area (SVL 38 mm) **D** All three species of rice paddy frogs found in Timor-Leste may show varying degrees of green coloration on the dorsal and lateral parts of the body. This specimen (SVL 56 mm) from the Viqueque area represents the most extreme green coloration we observed, in terms of both brightness and coverage. Photos by Mark O’Shea.

Rice paddy frogs (Fig. 4) are the most common amphibians found in regions with rice agriculture. They may grow to over 60 mm in snout-vent length and can usually be recognized by their fairly stout body shape, brownish to gray-green coloration, shiny moist skin with ill-defined dorsal and lateral patterns, and warts scattered irregularly or in rows along the back (e.g., Fig. 4B).

###### Collection and natural history.

As the name suggests, rice paddy frogs are commonly found in rice paddies where they perch at the water’s edge, on tufts of vegetation, or even on cow patties. Our survey documented at least three species of rice paddy frogs in Timor-Leste, with two or more often occurring in the same suitable habitat. Species can be distinguished by the size of mature males, which can be clearly separated into three groups by their size, morphology of the tympanum and its associated structures, and the patterning of the throat in males. One of the species, designated as *Fejervarya* sp. 1 [Ca CMD 431] is a lowland form and most similar to *Fejervarya verruculosa* (Roux 1910). This candidate species differs from *Fejervarya verruculosa* by the shape of the supratympanic fold, the size of the tympanum relative to the eye, patterning on the hidden surfaces of the legs, and number and position of maxillary teeth and the shape of the alary process of the premaxilla, among other characters. The other two candidate species can also be differentiated by these and other characters, and they have been designated *Fejervarya* sp. 2 [Ca CMD 508] and *Fejervarya* sp. 3 [Ca CMD 355].

Among the specific habitats where we encountered these frogs were active rice paddies, roadside puddles, coffee plantations, and coastal forests at altitudes between 4 m and 1187 m. Our observations are consistent with those of Menzies (1987) but we believe that not all three presumptive species are capable of such habitat plasticity. More detailed investigation is needed to clarify the habitat requirements of these three candidate *Fejervarya* species.

###### Biogeography.

The presence in Timor-Leste of three morphologically similar and seemingly endemic dicroglossid frogs, with an evolutionary origin in Asia, raises interesting biogeographic questions. The simplest explanation would be a single or a series of introductions in modern times, with the influx of peoples and cargo from points all across the Indonesian Archipelago. However, a more ancient, classic island biogeography scenario is also feasible. Based on the two main concepts of speciation, sympatric speciation among amphibians is possible but presumed rare among dicroglossid frogs. The concept of allopatric speciation is the alternative, by which the three species may represent descendants of three separate introductions that occurred as early farmers brought rice plants to the island. It is generally accepted that rice agriculture originally spread from China into South and Southeast Asia (Crawford and Chen 1998) and reached the Lesser Sunda Islands in waves after spreading throughout the Greater Sunda Islands during the Neolithic Period (Chi and Hung 2008). During this time, it is quite possible that stowaway frogs arrived on Timor with rice plants. The occurrence of several similar rice paddy frog species in sympatry is not unique to Timor-Leste (e.g., Burma; G. Zug, in litt.). Molecular studies to obtain some insights into this conundrum are progressing. The hypothesis that multiple human-mitigated introductions of *Fejervarya* populations occurred mirroring the development of rice cultivation is a plausible explanation for the many species of this genus listed as *incertae sedis* with respect to their intrageneric relationships.

##### Limnonectes timorensis

(Smith, 1927)

http://species-id.net/wiki/Limnonectes_timorensis

Fig. 5

###### Common names.

(E) Timor River Frog. *(T) Manduku mota (manduku = frog, mota = river).

###### Identification.

**Figure 5.**
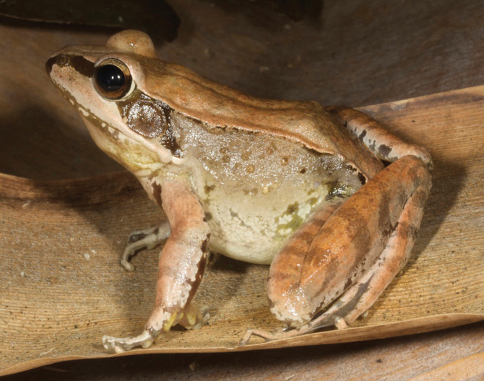
*Limnonectes timorensis*. Female from near Eraulo, Ermera District (USNM [CMD 422], SVL 62 mm). Photo by Mark O’Shea.

Frogs of the genus *Limnonectes* are nocturnal and generally quite difficult to distinguish from similar species. *Limnonectes timorensis* is currently the only known species of river frog recorded from Timor-Leste. Identifying characteristics include fingertips that are slightly swollen and widened at their tips, but which do not possess a marginal fold that outlines the disk pad. The first finger is invariably longer than the second. They possess a dorsolateral fold that originates just behind the eye and continues dorsally to the groin, and a tympanum that is nearly equal to the size of the eye (Fig. 5). A brown band is present on the head, arising near the tip of the snout, continuing along the canthus rostralis through the eye, and completely enveloping the tympanum. The skin warts commonly found concentrated on the dorsum in other species of *Limnonectes* are reduced in number on the dorsum but quite prominent on the side of the body (Fig. 5). We were readily able to confirm our identification by consulting the figure presented in Smith (1927: Pl. II, Fig. 1) and from the original description.

###### Variation.

Whereas in one of our specimens the internarial distance is slightly greater than the interorbital distance, a diagnostic characteristic provided by Smith (1927), the internarial distance is equal to the interorbital distance in the second. Even though Smith (1927) did outline some variation among his eight specimens, only slight differences in the interorbital distance are mentioned. It appears that the comparison of internarial distance with interorbital distance by itself is insufficient to distinguish this species from others.

In a second instance of incongruity between our specimens and the original description, the nares are not at the midway point between the eye and the tip of the snout but located approximately one third of the eye-to-snout distance away from the snout in both specimens. We believe that this incongruity could be due to an error in Smith’s (1927) description since the drawing of the frog (Smith 1927: Plate II, Figure 1) conforms to our specimens and not to Smith’s description. Scientific illustrators are generally extremely meticulous and accurate, and misplacement of the nares, a key character of the head, would be an unlikely error.

Lastly, the foot in our specimens is 7% longer than, as opposed to equal to, tibia length as described by Smith (1927). Repeated measurements with digital calipers and mechanical calipers as used in Smith’s day resulted in measurement errors of 3% and 7%, respectively (*n* = 10 for each instrument). We therefore believe that the discrepancy between foot and tibia length measurements we made on our specimens, and Smith made on his, is due to slight variation in the hindlimbs (Smith 1927:212), combined with measurement error, and does not represent a diagnostic difference.

###### Collection and natural history.

Two female individuals were collected during a single night from the Meleotegi River, near Eraulo, Ermera District, altitude 1179 m. During the dry season, the Meleotegi River is a relatively shallow stream that runs over pebbles and allows easy crossing. Boulders are distributed at irregular intervals along and in the riverbed. It is clear from the steeply eroded riverbanks (over 5 m high in some parts) that the river carries a large volume of water during parts of the year. One individual of *Limnonectes timorensis* was collected from a thin branch overhanging the relatively steep riverbank, whereas the other was found on a large boulder in midstream. No vocalizations were heard.

###### Taxonomic comment.

Forcart (1953) and Menzies (1987) considered *Limnonectes timorensis* a synonym of *Hylarana elberti* Roux 1911. Forcart’s (1953) synonymy was based on a comparison of specimens in the Naturhistorisches Museum Basel, Switzerland with the holotype of *Hylarana elberti* by Robert Mertens. One of us (HK) has made a very careful comparison of our *Limnonectes timorensis* specimens with the holotype of *Hylarana elberti*, and found differences in the tuberculation of the hand, the width of fingertips, the position and size of the rictal gland at the angle of the jaw, the extent of toe webbing, the width of the toe tips, and the patterning and consistency of the skin on the throat. Additional features of the shape of the head are difficult to compare because the holotype of *Hylarana elberti* has a damaged anterior of the head. We therefore agree with Dubois (1986) that the species from Timor and Wetar are distinct.

#### Family Hylidae – Treefrogs

##### Litoria everetti

(Boulenger, 1897)

http://species-id.net/wiki/Litoria_everetti

Fig. 6

###### Common names.

(E) Everett’s Timor Treefrog. *(T) Manduku ai Timor (manduku = frog, ai = tree).

###### Identification.

**Figure 6.**
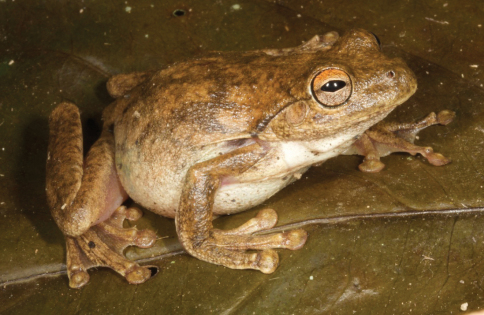
*Litoria everetti*. Female from near Eraulo, Ermera District (USNM [CMD 420], SVL 59 mm). Photo by Mark O’Shea.

*Litoria everetti* can easily be recognized by a combination of the following traits: webbed hands and feet, expanded finger and toe tips or disks, a well-developed supratympanic fold (Fig. 6). The hidden portions of the legs have an orange marbled pattern in life. In common with most treefrogs, this species is nocturnal. Menzies (1987) provided an excellent description of this species and a photograph. SJR is familiar with this species and could confirm its identity.

###### Collection and natural history.

Two females of *Litoria everetti* were collected at night at the same locality and during the same night as *Limnonectes timorensis*. Based on their overall morphology and characteristic webbing, as well as their characteristically orange marbled thighs (Boulenger 1897), these are clearly *Litoria everetti*. One specimen was found at the edge of the riverine embankment, perched on a branch. The second was caught on a small boulder near the water’s edge. *Litoria everetti* is a species in Red List Category “Least Concern” (Iskandar and Mumpuni 2004).

#### Family Rhacophoridae – Afro-Asian Foam-nest Treefrogs

##### Polypedates cf. leucomystax

(Gravenhorst, 1829)

http://species-id.net/wiki/Polypedates_leucomystax

Fig. 7

###### Common names.

(E) Striped Treefrog, Four-lined Treefrog, Golden Treefrog. (T) Manduku ai-riskadu (manduku = frog, ai = tree, riskadu = striped) or manduku loron (manduku = frog, loron = sunlight). (Fataluku) Nelu cila. The common names in Tetun and Fataluku are generalized name for treefrogs and may be applied to other such species without detailed distinction.

###### Identification.

**Figure 7.**
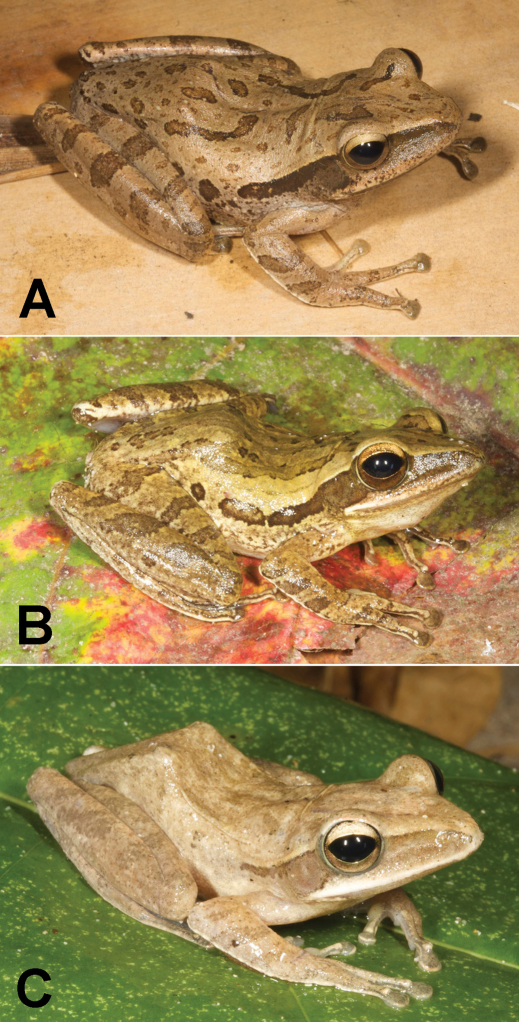
*Polypedates cf. leucomystax*. The individuals shown display the diversity of color patterns found in this species **A** A specimen from Bakhita (SVL 45 mm) displaying irregular dark brown spots and barred legs on a lighter brown background **B** A specimen from Loré, Lautém District (SVL 48 mm), presenting with a combination of brown dorsal and dorsolateral lines and leg barring on a nearly yellow background **C** A specimen (SVL 46 mm) from the same locality as **B**, showing a very lightly colored dorsum devoid of lines and spots. Photos by Mark O’Shea.

This species is a relatively slender treefrog with a variety of dorsal patterns (Fig. 7). The background coloration is usually a light brown during the nightly activity period but becomes a deeper brown while individuals are resting in their diurnal refuges. Patterning may consist of darker lines or bands, brown blotches, crossbars on limbs, or there may be no pronounced pattern. In comparison with the two other potential tree-dwellers encountered, *Limnonectes timorensis* and *Litoria everetti*, *Polypedates cf. leucomystax* is more slightly built, has a pointed snout, lacks raised warts or tubercles on the back, and has no webbing between the fingers.

###### Collection and natural history.

These frogs were quite commonly found, calling from the edges of ponds (e.g., 6.0 km W Loré 1 village, Lautém District), from small shrubs and bushes (e.g., 5 km S Malahara, Lautém District), from the trunks of fallen trees, and from boulders in the middle of streambeds (e.g., near Timor Village Hotel, Wailakurini, Viqueque District), at altitudes from near sea level to 1350 m at Maubisse, Ainaro District.

###### Taxonomic comment.

Our experience with the geographically widespread rhacophorid usually identified as *Polypedates leucomystax*, with a range extending from Borneo to Peninsular Malaysia, and from India to Cambodia, is that it is a taxon in need of closer investigation. On a recent trip we observed the species in Sabah, Borneo, and shortly thereafter in Timor-Leste. Based on vocalizations, behavior, maximum size, and color and pattern variation, we cannot confidently assign our specimens to *Polypedates leucomystax* and instead refer to them as *Polypedates cf. leucomystax*. The widespread morphotypes collectively known as *Polypedates leucomystax* are likely an assembly of similar rhacophorid species that occupy a treefrog niche in geographically distinct locations, as others have suggested (e.g., Narins et al. 1998). The taxonomic conundrum presented by *Polypedates leucomystax* is currently the subject of both molecular and morphological study (e.g., Brown et al. 2010), but a resolution has so far been elusive.

### Lizards (Order Lacertilia)

#### Family Agamidae – Agamas and Dragons

##### Draco timoriensis

Kuhl, 1820

http://species-id.net/wiki/Draco_timoriensis

Fig. 8

###### Common name.

(E) Timor Flying Dragon, Timor Flying Lizard. (T) Teki liras (teki = gecko, liras = winged). Fataluku: Lika. Mambae: Berdigil.

###### Identification.

**Figure 8.**
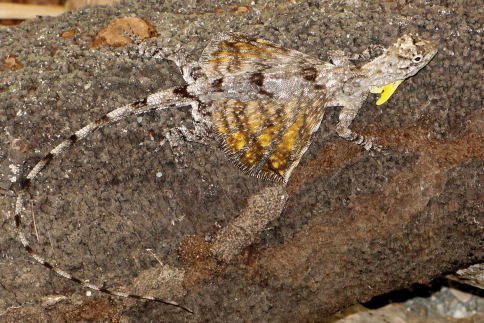
*Draco timoriensis*. Male from Wailakurini, Viqueque District (USNM 573658, SVL 75 mm, TL 208 mm). Photo by Hinrich Kaiser.

Lizards of the genus *Draco* are diurnal and easily identified by the presence of patagia. These ‘wing’ structures (Fig. 8) consist of skin flaps that are stretched across highly modified ribs that allow the lizards to glide between trees. Although referred to as ‘flying lizards’ these and other reptiles that have perfected this escape strategy are actually only gliding, flight being the preserve of birds, bats, and insects. They also possess a dewlap under the chin that males use for territorial display. *Draco timoriensis* is the only species of its genusknown to occur on Timor (see taxonomic comments below).

###### Collection and natural history.

We captured four specimens of *Draco timoriensis* and observed several others. All individuals were initially seen high off the ground (> 5 m) on the trunks or larger branches of trees but never on palm trees. Even though they are cryptically patterned against the bark background when stationary against the trunk of the tree, they are easily spotted when displaying their bright yellow dewlaps (Fig. 8). Our specimens were captured either using blowguns or by climbing the tree and forcing the lizard to glide to an accessible height. All specimens were seen and captured during the daytime. Where they occurred, these lizards were not rare. However, their dispersal pattern appears to be clumped (several lizards in one area with none outside of a particular territory) and we did not discern any pattern to their localized distribution. Based on our encounters, *Draco timoriensis* is limited in its distribution to altitudes from sea level to ca. 300 m.

###### Taxonomic comments.

Several historic reports of *Draco* collected on Timor list *Draco volans* or *Draco walkeri* in addition to *Draco timoriensis*. Based on recent unpublished findings from a molecular analysis (J. McGuire, in litt. 13 Oct 2009), *Draco volans* is confined to Bali and Java whereas the distribution of *Draco walkeri* is limited to Sulawesi (McGuire et al. 2007). All records for flying lizards from Timor should therefore be attributed to *Draco timoriensis*.

The species name of the Timor flying lizard has variously been spelled *timoriensis* or *timorensis*. In the accepted original description (Kuhl 1820:103), the name is given as *Draco Timoriensis* Péron. However, Péron never published a description of a *Draco* from Timor, even though the specimens from his expedition were presented to the Museum National d’Histoire Naturelle in Paris. The name *Draco Timoriensis* also appears in Duméril and Bibron (1837: 454), who list a manuscript by Péron first in their list of synonyms, with Kuhl (1820) relegated to second place. The first mention of the name “*timorensis*” is probably an unjustified emendation by Gray (1845), who listed the species as *Draco Timorensis* and referred to *Draco viridis Timorensis*, a plate in Schlegel (1837–44).Subsequent authors, beginning with Günther (1864) and Boulenger (1885), have perpetuated this error even though the latter corrected Gray in the spelling of the name attributed to Schlegel by listing it as *Draco viridis* var. *timoriensis*. Since this change in the spelling of the specific epithet is not via an accepted *nomen substitutum* (as suggested by Wermuth 1967), the correct spelling for the flying lizard found on Timor remains *Draco timoriensis*.

#### Family Gekkonidae – True Geckos

##### Genus Cyrtodactylus

http://species-id.net/wiki/Cyrtodactylus

Fig. 9

###### Common name.

(E) Timor Bent-toed Gecko. *(T) Teki ain-fuan kleuk (teki = small gecko, kleuk = bent, ain-fuan = toe).

###### Identification.

**Figure 9.**
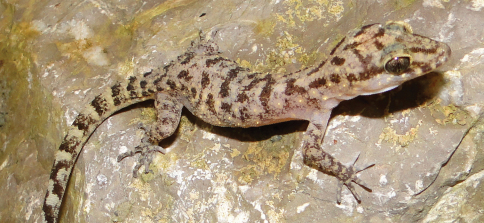
Undescribed species of *Cyrtodactylus*. Female from near Same, Manufahi District (USNM [CMD 383], SVL 58 mm, TL 127 mm). Photo by Hinrich Kaiser.

This candidate species of *Cyrtodactylus*, designated as *Cyrtodactylus* sp. 1 [Ca CMD 383], lacks the characteristic orange banding pattern of the tokay gecko (*Gekko gecko*) and has dorsal patterning with a greater amount of brown components (spots, flecks, lines) than any other gecko found on Timor. In its size, it is intermediate between the common house geckos (*Hemidactylus frenatus*, *Gehyra mutilata*) and the tokay gecko, and it does not have a flattened tail or dorsolateral skin flaps as in *Hemidactylus platyurus*. It is also the only gecko to possess non-dilated digits, unlike those found in typical geckos. Instead the toes are slender and curved (Fig. 9), resulting in various names being inconsistently applied to members of the genus (e.g., bent-toed geckos, naked-toed geckos, bow-fingered geckos). The genus *Cyrtodactylus* is the most diverse genus within the seven families comprising the Gekkota, with at least 130 species described.

###### Collection and natural history.

Two specimens of what is clearly an undescribed species of *Cyrtodactylus* were captured on the same night at the Trilolo River near Same, Manufahi District (altitude 553 m). There are substantial differences in pholidosis and overall morphology from all known species of *Cyrtodactylus* (see Rösler and Glaw 2008: Table 1). One individual was found on a boulder-face along the riverbank, while the second was in leaf litter at the foot of a large boulder at the boundary between riverine habitat and coffee plantation.

##### Gehyra cf. mutilata

(Wiegmann, 1834)

http://species-id.net/wiki/Gehyra_mutilata

Fig. 10

###### Common names.

(E) Mutilated Gecko, Stump-toed Gecko, Tender-skinned Gecko. *(T) Teki kulit kanek (teki = small gecko, kanek = injured, kulit = skin).

###### Identification.

**Figure 10.**
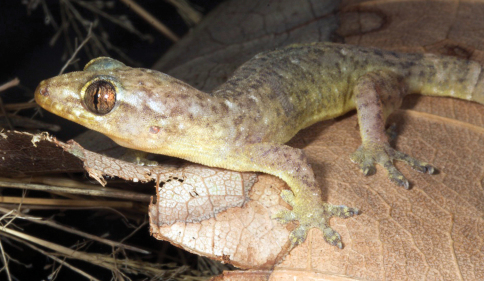
*Gehyra cf. mutilata*. Male (USNM [CMD 459], SVL 50 mm, TL 102 mm) from Loré 1 village, Lautém District. Photo by Mark O’Shea.

Individuals of the genus *Gehyra* (Fig. 10) in Timor-Leste are most commonly seen around human habitations, where they occur sympatrically with the common house gecko *Hemidactylus frenatus*. Identification on sight is usually quite difficult because of the superficial similarity of these two species. Upon capture, an early indication that a specimen is *Gehyra cf. mutilata* is its ability to shed skin and scales as a defensive mechanism. Unless great care is taken, the skin tears very easily at capture and the animal will appear ‘mutilated.’ Furthermore, the anterior and posterior postmental chin shields are elongate and in broad contact down the midline in *Gehyra mutilata*, whereas in *Hemidactylus frenatus* these chin shields are shorter, more rounded, and only the anterior pair is in midline contact, the posterior pair being widely separated by heterogeneous granular scales.

###### Collection and natural history.

The three specimens of *Gehyra cf. mutilata* we collected occurred syntopically with *Hemidactylus frenatus* and were invariably collected at the same time as specimens of that species. They occurred on the walls of houses as well as on the trunks of trees. It is possible that *Gehyra cf. mutilata* was introduced to Timor at some point during prehistoric human colonization or pre-colonial or colonial inter-island trade.

###### Taxonomic comment.

Even though *Gehyra mutilata sensu stricto* is a widely distributed species and occurs throughout Southeast Asia and the western Pacific realm, there is very little known about its exact distribution in Wallacea (Fisher 1997). There are several different names in the literature that could be applied to *Gehyra* populations on Timor that are not *mutilata*. Until we unequivocally confirm the identity of our specimens, they are here listed as *Gehyra cf. mutilata*.

##### Gekko gecko

(Linnaeus, 1758)

http://species-id.net/wiki/Gekko_gecko

Fig. 11

###### Common names.

(E) Tokay Gecko. (T) Toke.

The Tetun common names for geckos, teki (smaller geckos) and toke (the large tokay) are also used as slang meaning to identify young single women or men, respectively.

###### Identification.

**Figure 11.**
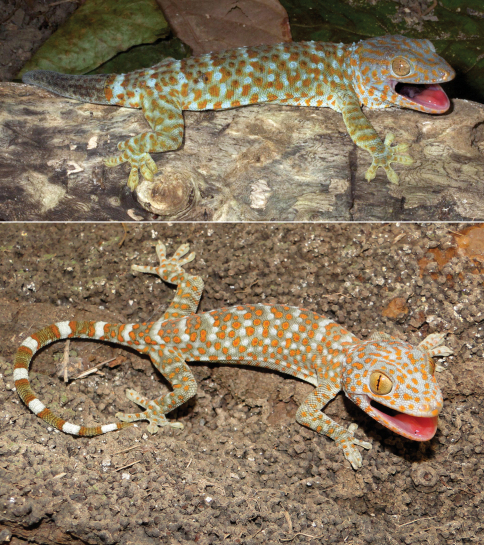
Tokay gecko (*Gekko gecko*). Adult male from Same, Manufahi District (USNM 573671, SVL 142 mm, TL 236 mm; top) and juvenile *Gekko gecko* from Wailakurini, Viqueque District (USNM 573673, SVL 88 mm, TL 168 mm; bottom). Note the brownish, regenerated tail on the adult (top). Photos by Mark O’Shea (top) and Hinrich Kaiser (bottom).

Tokay geckos are easily identified by their striking orange dorsal patterning (Fig. 11), as well as by their aggressive open-mouth display when encountering a threat. They also have a distinctive vocalization (“to-keh”) that gave them their common name. The dark-light banding pattern on the tail of hatchling *Gekko gecko* may at first glance be confused with a similar pattern on the tails of some bent-toed geckos (genus *Cyrtodactylus*). Based on overall habitat needs, if a gecko with a banded tail is encountered on the walls of human habitations in Timor-Leste, it is most likely *Gekko gecko*. Geckos of the genus *Cyrtodactylus* lack the dilated scansors necessary for climbing walls and are generally not associated with man-made structures.

###### Collection and natural history.

We found tokay geckos inhabiting nearly all of the hotels and guest houses in which we stayed, in addition to many other buildings and structures, as well as wooded regions in Lautém District (e.g., Loré 1 village). Tokay geckos were not present at the higher altitude localities we searched (above 1000 m). We collected one adult (Fig. 11 upper) and three juveniles (e.g., Fig. 11 lower) to secure vouchers and then discontinued the collection of this species. Individuals were observed preying on insects attracted by artificial light sources as well as on smaller geckos (e.g., *Hemidactylus frenatus*). One particularly aggressive individual even attacked a smaller gecko that we had stunned using a blowgun and placed into a plastic bag for safekeeping, and pulled it behind a bamboo wall inside one of our sleeping cabins. Eggs of what we presumed to be *Gekko gecko* from their size, and by the presence of adult tokays in the immediate vicinity of the clutches, were discovered in communal groups in rotting logs, on the walls of huts, and in tree holes. Based on their pattern of distribution and habits, it is possible that tokay geckos were introduced to Timor via inter-island trading or during colonization.

##### Hemidactylus frenatus

Schlegel, 1836

http://species-id.net/wiki/Hemidactylus_frenatus

Fig. 12

###### Common names.

(E) Common Indo-Pacific House Gecko. *(T) Teki uma baibain (teki = small gecko, uma = house, baibain = common).

###### Identification.

See comments under *Gehyra cf. mutilata*.

###### Collection and natural history.

This species is the most commonly encountered gecko in Asia, and it has also become an established exotic in many places in the New World. As a perianthropic species, it is present on the walls or among the rafters of almost every building, and it is distributed at widely differing altitudes and on the edges of many different habitats. This species is among the several gecko species found in Timor-Leste that may have been introduced during prehistoric colonization or historical inter-island trade. The species appeared absent from pristine habitats such as undeveloped forests, whereas it does occur in coffee plantations.

**Figure 12.**
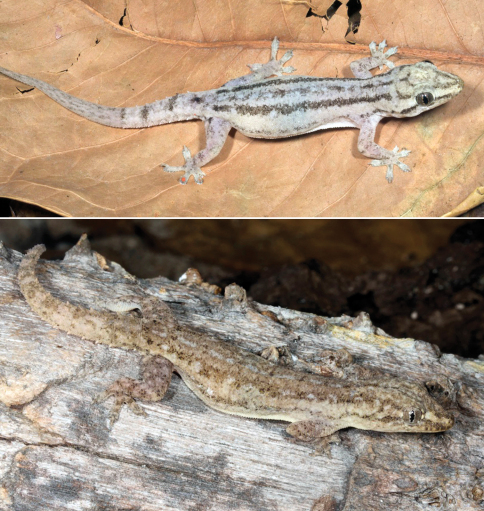
Individuals of *Hemidactylus frenatus* showing two distinctive color patterns. (Top) Specimen from near Baucau, Baucau District (USNM [CMD 526], SVL 47 mm, TL 90 mm) displaying a pattern of distinct dorsolateral stripes complemented by an interrupted, less distinct vertebral stripe. Note the regenerated tail and the bright orange mite infestation on the third toe, as well as an egg visible through the skin. (Bottom) Specimen from near Loré 1 village (USNM [CMD 488], SVL 42 mm, TL 89 mm) with a cryptic dorsal pattern. Photos by Mark O’Shea.

##### Hemidactylus platyurus

(Schneider, 1792)

http://species-id.net/wiki/Hemidactylus_platyurus

Fig. 13

###### Common names.

(E) Common Flat-tailed Gecko. *(T) Teki ikun belar (teki = small gecko, belar = flat, ikun = tail).

###### Identification.

**Figure 13.**
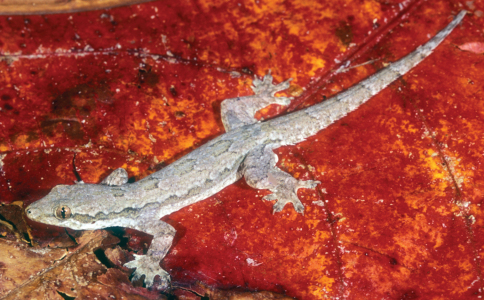
*Hemidactylus platyurus*. Male from Loré 1 village, Lautém District (USNM [CMD 458], SVL 41 mm, TL 91 mm). Photo by Mark O’Shea.

Flat-tailed geckos can be identified by the presence of lateral skin flaps and a flattened tail bearing a fringe of denticulate skin (Fig. 13). Such adaptations provide an increased measure of cryptic morphology for these geckos in addition to their bark-like coloration, as the skin extends almost seamlessly from body to substrate and all but eliminates any shadow these animals may cast.

###### Collection and natural history.

We collected several specimens by night when they were exposed on the trunks of trees or under bark in forested habitats at elevations of 300 m or less. In one instance, a pair was found in close proximity on the same tree. Geckos like these may also be found in a perianthropic setting or in lowland savannas, where they may be sympatric with *Hemidactylus frenatus* and *Gehyra cf. mutilata*. As these other two species, it is likely that *Hemidactylus platyurus* is not native to Timor but was introduced by human activities in the past.

###### Taxonomic comment.

Until the revision of geckos in the genus *Hemidactylus* by Carranza and Arnold (2006), this species was called *Cosymbotus platyurus*.

#### Family Scincidae – Skinks

##### Genus Carlia

http://species-id.net/wiki/Carlia

Fig. 14

###### Common names.

(E) Four-fingered Skinks. *(T) Mamór liman-fuan haat (mamór = skink, haat = four, liman fuan = finger).

###### Identification.

Skinks of the genus *Carlia* are small lizars that are often found foraging in grassy vegetation or under decaying palm fronds. Their identifying characteristic is a four-fingered forefoot – all other lizards in Timor-Leste possess five-fingered forefeet. In common with all other lizards, they possess pentadactyl hindfeet. Identification within the genus *Carlia* is often difficult and involves scale counts, color patterns, morphometrics, and natural history characters. George Zug (USNM) preliminarily verified the initial division of our specimens into groups.

###### Collection and natural history.

**Figure 14.**
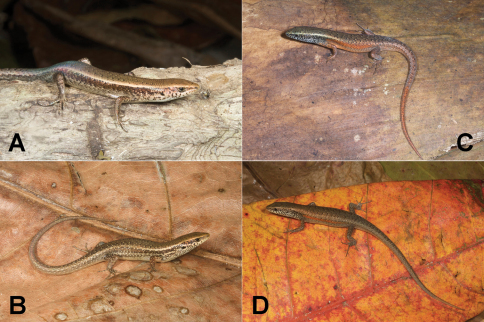
Representative specimens of the four presumed species of *Carlia* found in Timor-Leste. **A** *Carlia* sp. 1, a high-altitude form from Maubisse, Ainaro District (USNM [CMD 361], SVL 37 mm, TL 99 mm) **B** *Carlia* sp. 3, a lowland form from near Loré, Lautém District (USNM [CMD 483], SVL 43 mm, TL 108 mm) **C** *Carlia* sp. 2, a highland form from the western versant of Mt. Ramelau in Ermera District (USNM [CMD 401], SVL 44 mm, TL 112 mm) **D** *Carlia* sp. 4, a lowland form from near Baucau, Baucau District (USNM [CMD 522], SVL 42 mm, TL 112 mm). Photos by Mark O’Shea.

We encountered four phenotypically distinct four-fingered skinks of the genus *Carlia* (Fig. 14) throughout our survey. Considering how morphologically conservative species in this genus are, we are as yet unable to assign them to existing taxa with confidence. Comparisons with specimens of *Carlia peronii*, *Carlia spinauris*, and *Carlia fusca* (including the holotypes of *Carlia peronii* and *Carlia fusca* and a syntype of *Carlia spinauris*) show that the groups we found in Timor-Leste are similar to the former based on overall body morphology and size. There are, however, differences with *Carlia peronii* and *Carlia spinauris* (fide Zug 2010) that require further study. We acknowledge that a collection of *Carlia* in a relatively short time span may not allow us to assess the true breadth of morphological variation since coloration may depend on the degree of sexual maturity or reproductive readiness (G. Zug, pers. comm.). A more detailed morphological and genetic analysis is therefore underway.

Among our *Carlia* specimens, we can differentiate two high-altitude forms and two low-altitude forms. One of the high-altitude forms from the Maubisse and Same areas (Ainaro and Manufahi District, respectively) on the eastern slopes of Mt. Ramelau (altitudes 600–1500 m) resembles species in the *Carlia peronii* group (*sensu* Zug 2010), and we believe that this form may have previously been listed in the literature as *Carlia peronii* (e.g., Greer 1976). Based on our fresh material and on comparisons with preserved material, we are more comfortable with listing this form as *Carlia* sp. 1 [Ca CMD 354] (Fig. 14A) until we have completed a survey of material in herpetological collections. Similarly, a second high-altitude form from the western versant of Mt. Ramelau in Ermera District (altitudes near 1200 m) is also similar to species in the *Carlia peronii* group, and the orange ventral coloration of sexually mature individuals makes it readily distinguishable (Fig. 14C). At this time, we prefer to list this form as *Carlia* sp. 2 [Ca CMD 400] until additional work is completed. This form was found exclusively during the daytime and near dusk, in clearings at the edge of coffee plantations, amongst scattered piles of bamboo husks, and on grassy areas surrounding bamboo stands. Several individuals were also found in a pile of construction debris (metal piping and wooden boards). We noted the absence of *Carlia* specimens in open spaces with no nearby form of cover (as measured in a few lizard body lengths).

The two lowland forms are not easily placed in either of the above groups. Even though their morphology is conservative, there are differences in overall *gestalt* and coloration. Since our collections were conducted during what is considered a mainly dry time of the year, we may see some changes when we return during the wet season, as individuals may change color as they mature or reach reproductive readiness. At this point, we consider the form from the coastal dry forest near Loré (Lautém District) as *Carlia* sp. 3 [Ca CMD 471] (Fig. 14B). The collection of a single specimen from the Baucau area provides insufficient material to determine with clarity what its specific status should be, but given its distinct morphology and pending the collection of additional material we consider this form as *Carlia* sp. 4 [Ca CMD 522] (Fig. 14D). At the Loré site, individuals were most predictably found by turning over decaying palm fronds and in the leaf litter. In a typical display of skink behavior, *Carlia* were seen each morning, basking, hunting, or displaying in the various sunspots near our campsite.

##### Cryptoblepharus leschenault

(Cocteau, 1832)

http://species-id.net/wiki/Cryptoblepharus_leschenault

Fig. 15

###### Common name.

(E) Leschenault’s snake-eyed skink. *(T) Mamór matan samea (mamór = skink, matan = eye, samea = snake).

###### Identification.

The defining characteristic of this genus, which occurs widely across the tropical world, is the lack of moveable eyelids, which are replaced by transparent snake-like brilles. Their slender body, long tail, and distinctive dorsal pattern, consisting of two light dorsolateral stripes and a characteristic light mid-dorsal line that forks posterior to the neck, easily identifies individuals of this species. Our identification of this species was confirmed by reviewing the figures in Horner (2007: Figs. 170, 171).

###### Collection and natural history.

We collected four individuals of *Cryptoblepharus leschenault* and observed many others. These skinks were invariably seen on the trunks of hardwoods above root level. Two species of *Cryptoblepharus* have been reported from Timor, but we did not encounter *Cryptoblepharus schlegelianus*, which can be distinguished from *Cryptoblepharus leschenault* by a greatly reduced degree of dorsolateral striping, particularly the lack of a vertebral stripe that extends along the dorsum and onto the tail (Horner 2007: Fig. 188).

###### Taxonomic comment.

Salomon Müller first mentioned the occurrence of this species on Timor in letters written in 1829 (Brongersma 1942). Müller reported on collections he made on Semau, a small island off the northwest coast of West Timor, as well as on Timor. Müller’s descriptions include *Cryptoblepharus schlegelianus* and *Cryptoblepharus leschenault* and it appears that both of these species were collected within a short time span during his visits. Based on the specimens available to Brongersma in the 1940s in the collections at the RMNH (see Brongersma 1942) and our own search of herpetological collections, there exists a single voucher specimen of *Cryptoblepharus leschenault* from Timor in the collection of the Naturhistorisches Museum Basel (NHMB 12885, as *Ablepharus boutonii leschenault*), which was first reported by Forcart (1953). Our specimens therefore comprise the first series of *Cryptoblepharus leschenault* from Timor and Timor-Leste. Specimens collected of this species by Max Weber (see Weber 1890) were from Flores (described as *Ablepharus boutonii furcata*).

**Figure 15.**
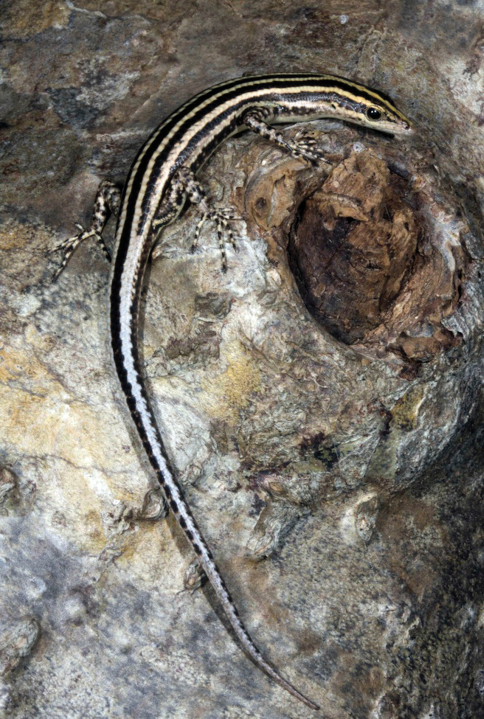
*Cryptoblepharus leschenault*.Male (USNM 573654, SVL 37 mm, TL 84 mm) from near Loré, Lautém District. Photo by Mark O’Shea.

##### Genus Eremiascincus

http://species-id.net/wiki/Eremiascincus

Fig. 16

###### Common names.

(E) Night Skinks. *(T) Mamór kalan (mamór = skink, kalan = night).

###### Identification.

Lizards of the genus *Eremiascincus* are relatively slender, long-bodied skinks with rounded, elongate, conical tails and reduced limbs. On Timor it is necessary to distinguish between at least five species of *Eremiascincus* (*Eremiascincus timorensis*, *Eremiascincus antoniorum*, *Eremiascincus emigrans*, *Eremiascincus* sp. 1 [Ca CMD 365], *Eremiascincus* sp. 2 [Ca CMD 474]), and the distinctions between these are rather finite.

###### Collection and natural history.

**Figure 16.**
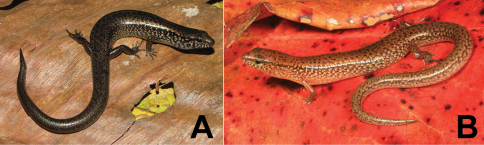
Individuals of two presumed undescribed species of *Eremiascincus*. **A** *Eremiascincus* sp. 1 (USNM [CMD 365], SVL 66 mm, TL 185 mm) from Maubisse, Ainaro District **B** *Eremiascincus* sp. 2 (USNM [CMD 474], SVL 51 mm, TL 101 mm) from Loré 1 village, Lautém District. Photos by Mark O’Shea.

We collected two of the species of *Eremiascincus* found on Timor. *Eremiascincus* sp. 1 (Fig. 16A) is a species whose distribution is apparently limited to elevations above 1000 m. Individuals were primarily found under logs and rocks, and were never encountered in the open during the day. *Eremiascincus* sp. 1 was the highest-altitude reptile we recorded in Timor-Leste (southwestern slopes of Mt. Ramelau, 2046 m). We found three individuals of *Eremiascincus* sp. 2 (Fig. 16B) among fallen and decaying palm fronds near Loré 1 village, Lautém District. These individuals were found syntopically with many individuals of *Carlia* sp. 3. Their activity level was highest just before dusk.

###### Taxonomic comment.

In their recent comprehensive molecular study of desert skinks (Australian members of *Eremiascincus* Greer, 1979), Mecke et al. (2009) revealed that several species of the polyphyletic genus *Glaphyromorphus* Wells and Wellington 1983, including the *isolepis* group *sensu* Greer (1990), required reassignment to *Eremiascincus*. This expands the definition of *Eremiascincus*, previously a genus comprised entirely of desert forms, to one including tropical species. For these species, which includes all named species from Timor as well as the candidate species listed herein, we use the term night skinks since they appear to adopt a more nocturnal or crepuscular activity cycle, in contrast to most other skink species.

##### Eutropis cf. multifasciata

(Kuhl, 1820)

http://species-id.net/wiki/Eutropis_multifasciata

Fig. 17

###### Common names.

(E) Common Sun Skink, Many-lined Sun Skink. *(T) Mamór loro (mamór = skink, loro = sun).

###### Identification.

**Figure 17.**
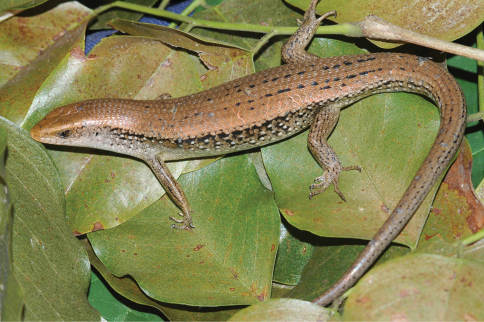
*Eutropis cf. multifasciata*. Male (not captured, TL ca. 225 mm) from Lospalos, Lautém District. Photo by Stephen Richards.

Skinks of the genus *Eutropis* are among the most robust lizards in Timor-Leste, following in size only *Varanus timorensis* and *Gekko gecko*. They can be identified by their brown dorsal coloration, smooth scales, and brownish black lateral blotches (Fig. 17).

###### Collection and natural history.

We collected two specimens of a very robust form of *Eutropis* that closely resembles *Eutropis multifasciata*. Because of the limited size of our sample and because of observable differences between specimens from Timor-Leste and those from other parts of Southeast Asia, we report these specimens as *Eutropis cf. multifasciata* pending a more thorough morphological and genetic analysis. Both specimens were collected during the day while foraging in grassy vegetation.

###### Taxonomic comment.

Until the genus *Eutropis* was proposed for Asian members of the former circumtropical scincid genus *Mabuya* (Mausfeld and Schmitz 2003), this taxon was known as *Mabuya multifasciata*.

##### Lamprolepis cf. smaragdina

(Lesson, 1826)

http://species-id.net/wiki/Lamprolepis_smaragdina

Fig. 18

###### Common names.

(E) Emerald Tree Skink. *(T) Mamór modok (mamór = skink, modok = green).

###### Identification.

In individuals of *Lamprolepis cf. smaragdina* that possess the name-giving color pattern, identification is easy. There are no other lizards reported from Timor whose anterior body coloration is an emerald green. Individuals that lack this color pattern may be confused with *Eutropis cf. multifasciata*, although in a direct comparison the darker dorsal coloration and lateral spotting, along with stouter body proportions, of the latter should be diagnostic. Individuals of *Lamprolepis* are generally found on the upper portion of the trunk of trees and palms, just below the foliage or crown, into which they will quickly retreat when disturbed during basking, whereas individuals of *Eutropis* were not observed on trunks at all and will retreat into grassy areas or under ground-level cover.

###### Collection and natural history.

The several specimens of *Lamprolepis cf. smaragdina* we collected were taken from the trunks of trees by blow-piping or hand-collecting. Several individuals were observed basking in sunspots very close to a specific tree, to which they retreated when disturbed. A retreat would usually occur in stages, first by climbing the trunk of the apparent ‘home tree’ to just below the tree’s foliage while the skink maintained visual contact with the intruding human and then, when the threat persisted, a total retreat into the dense foliage or crown of the tree or palm. We were able to find what we believe to be the same individuals of *Lamprolepis cf. smaragdina* on the same tree during several days of observation, indicating that these lizards display strong site fidelity.

**Figure 18.**
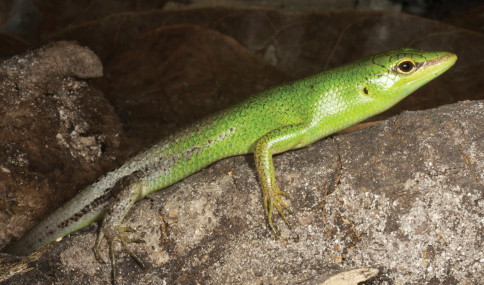
*Lamprolepis cf. smaragdina*. Male (USNM 573669, SVL 92 mm, TL 216 mm) from Loré 1 village, Lautém District. Photo by Mark O’Shea.

Unlike the entirely emerald green *Lamprolepis smaragdina* we have encountered elsewhere (HK in Peninsular Malaysia, MOS and SJR in Papua New Guinea), the solid bright green coloration of Timor-Leste specimens was limited to the anterior half of the body, posterior to which it morphed into a beige brown with dark dorsal spots and dark lateral striping (Fig. 18). Two of our specimens lacked any green coloration and sported a beige brown, pepper-and-salt patterned dorsal coloration. Coloration was, surprisingly, not sexually dichromatic, and among the two males and two females in our collection both sexes are represented by one bicolor green and brown specimen and one entirely brown specimen.

###### Taxonomic comments.

The pepper-and-salt color pattern we observed in our specimens is reminiscent of the patterns described for *Lamprolepis s. moluccarum* by (Barbour (1911, 1912) and for *Lamprolepis s. elberti* by Sternfeld (1920). We defer the decision on the exact taxonomic allocation of our specimens until a more detailed comparison, to include the named Wallacean subspecies of *Lamprolepisv smaragdina*, has been conducted.

##### Genus Sphenomorphus

http://species-id.net/wiki/Sphenomorphus

Fig. 19

###### Common names.

(E) Wedge skinks. *(T) Mamór ai laran (mamór = skink, ai laran = forest).

###### Identification.

**Figure 19.**
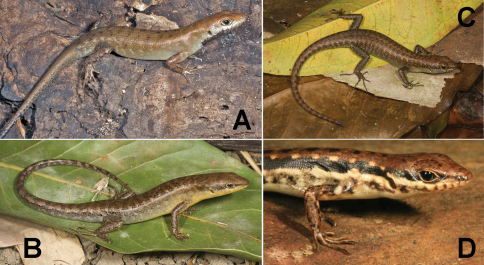
Representative examples of the four putative undescribed species of wedge skinks, genus *Sphenomorphus*.**A** *Sphenomorphus* sp. 1 (USNM [CMD 446], SVL 58 mm, TL 150 mm) from Loré, Lautem District **B** *Sphenomorphus* sp. 2 (USNM [CMD 364], SVL 66 mm, TL 185 mm) from Maubisse, Ainaro District **C** *Sphenomorphus* sp. 3 (USNM [CMD 416], SVL 69 mm, TL 152 mm) from the Meleotegi River near Eraulo, Ermera District **D** *Sphenomorphus* sp. 4 (USNM [CMD 415], SVL 42 mm, TL 92 mm) from the same locality as the animal in **C** Photos by Mark O’Shea.

The genus *Sphenomorphus* includes wedge skinks of greatly varying sizes and diverse morphologies. The superficially conservative morphology of these skinks is contradicted by a significant number of differences in the details of scalation and coloration. Beyond a recognition based on color pattern (as is straightforward for the species shown in Fig. 19D), these forms are difficult to tell apart. *Sphenomorphus* sp. 1 [Ca CMD 445] (Fig. 19A) possesses a series of paired dark paravertebral spots running as two lines onto the tail. In both sexes, the ventral coloration is cream, and males possess a black throat. *Sphenomorphus* sp. 2 [Ca CMD 356] (Fig. 19B) has a more diverse pattern of spots on its back, including brown, golden, and black spots in a complex arrangement. The throat is not black in males, and in both sexes the venter is yellow. *Sphenomorphus* sp. 3 [Ca CMD 415] (Fig. 19C) has a dorsal color pattern that is more uniformly brown, with some transverse golden dorsolateral striping. The venter of both sexes is a dirty cream color. *Sphenomorphus* sp. 4 [Ca CMD 416] (Fig. 19D) is easily differentiated from the other forms by its smaller size and by a characteristic black lateral stripe that extends from the eye along the side of the body all the way to the tip of the tail. Its dorsal coloration is more reddish brown than that of the other forms.

###### Collection and natural history.

We collected four forms of *Sphenomorphus* at three very distinct localities. In the area around Maubisse at altitudes >600 m, we encountered a highland form (*Sphenomorphus* sp. 2; Fig. 19B) that frequently shared its hiding places with night skinks (*Eremiascincus*). A worker at a road construction site gave to us one specimen smaller than typical individuals of *Sphenomorphus* sp. 2 but of a very similar morphology. That lizard was already injured from rough handling and expired shortly after we received it. Based on the surrounding vegetation, the altitude (>600 m), and several morphological features, we refer this specimen to *Sphenomorphus* sp. 2 pending the collection of additional specimens and a more thorough analysis. There is superficial resemblance of *Sphenomorphus* sp. 2 to *Sphenomorphus variegatus* (Peters 1867), but a further evaluation of museum specimens is necessary to verify any species assignment.

At a second highland locality, in the area of Eraulo (Ermera District) on the western side of the Mount Ramelau massif, we found two distinct forms of *Sphenomorphus*. One of these, from the Meleotegi River, is a form with very distinctive dorsal patterning (Fig. 19D). It was discovered while turning over flat rocks at the edge of the river. Based on its morphology we document this form as *Sphenomorphus* sp. 4. In the adjacent forest and plantation habitats we collected two specimens of *Sphenomorphus* sp. 3 (Fig. 19C).

The lowland form (Fig. 19A) from the dry coastal forest at Loré (Lautém District) is quite common throughout the habitat. Individuals are most easily found during the daytime on the buttresses and roots of trees or whilst foraging in the leaf litter. This form has strong resemblance to *Sphenomorphus florensis* (Weber 1890), but since that species currently has three subspecies aside from the nominate form (Dunn 1927: *Sphenomorphus f. nitidus*, * f. barbouri*, *Sphenomorphus f. weberi*) additional comparative work is necessary to determine its exact species affinity. It is also possible that the taxonomy is complicated by the possible synonymy of *Sphenomorphus florensis* and *Sphenomorphusmelanopogon* (Duméril and Bibron 1839); one of the syntypes of *Sphenomorphus melanopogon* reportedly was collected on Timor. At this time, we prefer to call this form *Sphenomorphus* sp. 1 (Fig. 19A).

###### Taxonomic comments.

In her seminal work on the reptiles of the Indo-Australian region, Nelly de Rooij (1915) provided species accounts for *Lygosoma florense* and *Lygosoma variegatus*, having examined specimens of only the latterfrom Timor. Based on our own examination of various type specimens, the Lesser Sunda species of the genus *Sphenomorphus* require careful additional investigation in order to confirm their species status and distribution. A revision of *Sphenomorphus florensis* is currently being conducted by Glenn Shea (in litt.).

#### Family Varanidae – Monitor Lizards

##### Varanus timorensis

Gray, 1831

http://species-id.net/wiki/Varanus_timorensis

Fig. 20

###### Common names.

(E) Timor Tree Monitor, Spotted Tree Monitor. (T) Lafaek rai-maran (lafaek = crocodile or large lizard, rai = dirt, maran = dry). (Mambae) Loti. (Fataluku) Puilolon.

###### Identification.

**Figure 20.**
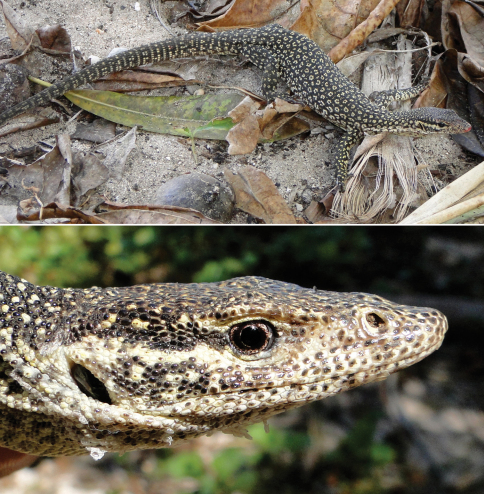
*Varanus timorensis*. Two adult males from dry coastal forest in Loré, Lautem District. Photos by Mark O’Shea (top) and Hinrich Kaiser (bottom).

Timor tree monitors are the largest lizards reported from Timor-Leste. Their identifying color pattern consists of circular yellow ocelli that cover the entire dorsum (Fig. 20 Upper). These lizards have nares that are positioned posterior to the snout by nearly a third of the distance from eye to the tip of the snout (Fig. 20 Lower), whereas they are positioned in close proximity of the snout in other lizards on Timor.

###### Collection and natural history.

We captured, photographed, and released four individuals of this small monitor lizard in the Loré area, Lautém District, and two in the Tutuala Beach area (Pantai Walu). One individual was seen repeatedly on a roadside retaining wall in Tutuala just west of the turnoff for Tutuala Beach. Another individual was observed basking in a sunspot in coastal dry forest from where it escaped into the hole of a dead branch. Three individuals were active in the undergrowth in coastal dry forest. Two individuals were hiding by night under loose bark on a tree and could initially only be recognized by their exposed tails. We have also seen road-killed specimens of this species on roadways running through rice paddies.

While driving along the coast road of northern Timor-Leste, we caught glimpses of monitor lizards of uncertain species affinity crossing the ‘blacktop.’ On at least one occasion we were able to ascertain by visual identification that the individual was *Varanus timorensis*, but on other occasions we could not make a positive identification. *Varanus indicus*, a widespread Indo-Pacific species of monitor lizard with similar body aspect and lifestyle to *Varanus timorensis*, has been reported from Timor, although we have so far been unable to find any corroboration, either via photography, voucher specimen, or artistic representation, that *Varanus indicus* is present on Timor. Such reports may stem from fleeting identifications by visitors familiar with the *Varanus indicus* body morphology. Reports from local villagers regarding the presence of a large monitor lizard similar to *Varanus salvator* from swamps in the Becora area require investigation.

### Snakes (Order Serpentes)

Our current treatment of the snakes of Timor-Leste is primarily restricted to the land-inhabiting species. Exceptions are those species, such as sea kraits (*Laticauda*) or filesnakes (genus *Acrochordus*), whose lifestyle frequently places them on land or into land-associated aquatic ecosystems. However, eleven species of seasnakes (Acalyptophis peronii*, Aipysurus apraefrontalis*, Aipysurus duboisii*, Aipysurus foliosquama*, Aipysurus fuscus*, Aipysurus laevis*, *Astrotia stokesii*, Emydocephalus annulatus*, *Hydrophis melanocephalus*, *Lapemis hardwickei*, *Pelamis platurus*)have been collected from the reefs of the Sahul Shelf to the southwest of Timor (Hibernia, Ashmore, Cartier and Scott Reefs; Minton and Heatwole 1975). One of us (MOS) visited Hibernia and Ashmore Reefs in 2001 and recorded the seven species marked by asterisks above, although *Aipysurus apraefrontalis*, *Aipysurus foliosquama*, and *Aipysurus fuscus* are believed endemic to the reefs. The other species may easily occur along the coasts of Timor, on coral reefs, in estuaries or in coastal waters, as may species distributed further east on the Australian and New Guinea coasts, or further north in the Banda or Flores Seas.

#### Family Colubridae – Typical Snakes

##### Coelognathus subradiatus

(Schlegel, 1837)

http://species-id.net/wiki/Coelognathus_subradiatus

Fig. 21

###### Common names.

(E) Timor Racer, Lesser Sunda Racer, Lesser Sunda Trinket Snake. *(T) Samea laho (samea = snake, laho = rat).

###### Identification.

**Figure 21.**
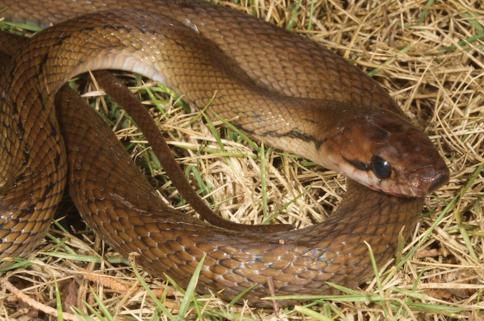
*Coelognathus subradiatus*. Male from Baucau town, Baucau District (USNM 573676). Photo by Mark O’Shea.

*Coelognathus subradiatus* is a slender brown racer that can be identified by a pair of black paravertebral stripes that run along its back from the rear of the head to the tail, with varying degree of completeness (Fig. 21). This snake also has a short (1–3 scales) longitudinal postorbital stripe and a flat, squared tip to the snout.

###### Collection and natural history.

One individual was captured at night on the road leading down from Old Town Baucau into the surrounding lowlands, but still in the outskirts of the city (altitude 350 m). It had been hit by another vehicle and succumbed to its injuries shortly after collection. *Coelognathus subradiatus* populations in Timor-Leste may not be conspecific with those on Roti, a neighboring island to the west, or from some of the other Lesser Sunda Islands (Monk et al. 1997).

###### Taxonomic comment.

We follow the taxonomy of Schulz (1996) who places the populations of *Coelognathus subradiatus* from Timor, the type locality according to the description of specimens by Schlegel (1873–44) collected there by Salomon Müller, in his Group 1. Of interest is that a subspecies of *Coelognathus subradiatus* was designated by Bethencourt Ferreira (1897) for a population from Timor, described as “*Coluber melanurus* (Schl.), var. *timoriensis*, n. var.” from specimens in the Museo Bocage in Lisbon. Whereas *Coluber melanurus* Schlegel 1837 is currently known as *Coelognathus flavolineatus*, Bethencourt Ferreira’s (1897, 1898) detailed description clearly allows identification of the Lisbon specimens as *Coelognathus subradiatus*. We believe that in his description, and lacking a familiarity with actual specimens of *Coelognathus subradiatus* from other collections, (Bethencourt Ferreira (1897, 1898) erred on the side of caution by identifying his specimens as a unique variant related to *Coluber melanurus* and young *Coluber erythrurus* (Duméril et al. 1854). Bethencourt Ferreira’s accounts were apparently cited only twice in relation to this snake population, first by Barbour (1912) and then by de Rooij (1917). Without comment, Barbour (1912: 195) lists this population as a full species (*Elaphe timoriensis*) in his expansive table, considered an error by Schulz (1996). In an unfortunate set of circumstances, this confused situation has been compounded by the complete loss of specimens and collection data in the Museo Bocage by fire in 1978. Until the revision of Old World ratsnakes by Helfenberger (2001), this species was placed in the genus *Elaphe*.

##### Dendrelaphis inornatus timorensis

(Smith, 1927)

http://species-id.net/wiki/Dendrelaphis_inornatus_timorensis

Fig. 22

###### Common names.

(E) Timor Bronzeback, Timor Treesnake. *(T) Samea kotuk kór kafé (samea = snake, kotuk = back, kór kafé = brown).

###### Identification.

**Figure 22.**
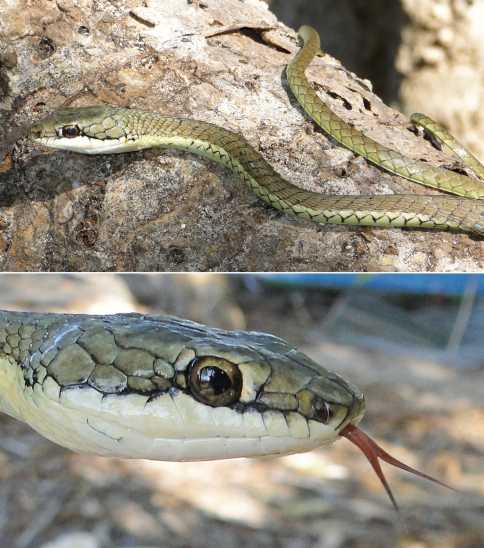
*Dendrelaphis inornatus timorensis*. Male (USNM [CMD 493], SVL 689 mm, TL 1054 mm) from Loré 1 Village, Lautém District. Photos by Hinrich Kaiser.

Bronzebacks are slender, diurnal snakes capable of rapid arboreal locomotion that may confuse the eye. The Timor bronzeback is brown above and with a greenish cream venter (Fig. 22 Upper). A narrow black stripe separates the dorsum of the head from the paler labial scales of the mouth (Fig. 22 Lower). When threatened, bronzebacks may inflate their neck, exposing the blue interstitial skin between their scales and making themselves look larger and more threatening to potential attackers. Identification and comparison with the widespread common bronzeback *Dendrelaphis pictus* was made in accordance with How et al. (1996) and David and Vogel (1996) and was verified by Gernot Vogel. The taxonomy of *Dendrelaphis inornatus* is currently undergoing a re-evaluation by Vogel and Jan van Rooijen (Gernot Vogel, pers. comm.).

###### Collection and natural history.

We collected three specimens of this subspecies (one adult, two juveniles). Two were found at night in a resting position in shrubs or bushes no higher than 2 m off the ground. These sleeping snakes became alert once illuminated by our flashlights, and they attempted to escape thereafter. The third specimen was observed while it travelled through the leaf litter in dry coastal forest. A fourth specimen was seen in the proximity of the third, but on the trunk of the tree. When pursued, this snake rapidly ascended the trunk and disappeared in the foliage.

##### Lycodon capucinus

(Boie, 1827)

http://species-id.net/wiki/Lycodon_capucinus

Fig. 23

###### Common names.

(E) Common Wolfsnake. (T) Samea lobo (samea = snake, lobo = wolf).

###### Identification.

**Figure 23.**
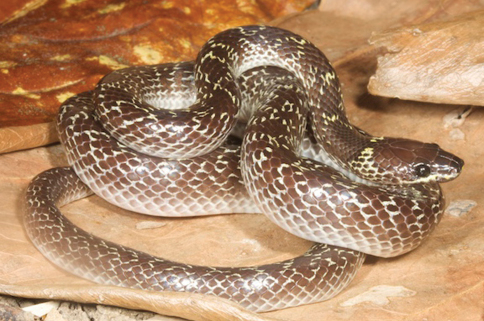
*Lycodon capucinus*. Male (USNM 573681, SVL 395 mm, TL 491 mm) from the town of Same, Manufahi Distict. Photo by Mark O’Shea.

Common wolfsnakes have a dorsally brown body with a series of weak pale yellow to white bands (Fig. 23). The dorsal part of the head is uniformly brown, offset from the rest of the body by a pale yellow nuchal band. The labial scales and venter are cream colored.

###### Collection and natural history.

Our single specimen of *Lycodon capucinus* was collected by local people in the town of Same (Manufahi District) while crossing the town’s main road after a heavy rain. It was brought to us undamaged in a 500-ml clear plastic water bottle.

###### Taxonomic comment.

In the *Lycodon* literature the names *Lycodon aulicus* and *Lycodon capucinus* are seemingly used interchangeably, sometimes with *capucinus* relegated to subspecific status within *aulicus*. During the time when *capucinus* had subspecific status, some authors did not differentiate it from *aulicus sensu stricto*. When *capucinus* was controversially re-elevated to specific status, this compounded an already confusing situation. As a consequence, the *Lycodon* forms from Southeast Asia and Wallacea under consideration here have been known by three possible species and subspecies names. We here follow Taylor (1965) and David and Vogel (1996) in using the name *Lycodon capucinus* for the Lesser Sunda form.

##### Lycodon subcinctus

Reinwardt, 1827

http://species-id.net/wiki/Lycodon_subcinctus

Fig. 24

###### Common names.

(E) Malayan Banded Wolfsnake. *(T) Samea kadeli (samea = snake, kadeli = ring).

###### Identification.

**Figure 24.**
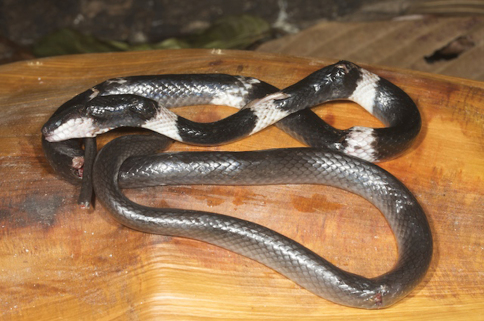
*Lycodon subcinctus*. Damaged specimen (USNM 573682, SVL 544 mm, parts of tail lost) from Letefoho, Manufahi District. Photo by Mark O’Shea.

Banded wolfsnakes are easily identified by the series of contrasting white to cream-colored bands that offset the dark brown to black body coloration (Fig. 24). In this coloration, they mimic Malayan or many-banded kraits (*Bungarus candidus* and *Bungarus multicinctus*, respectively), highly venomous species, with which this wolfsnake is sympatric in northern parts of its range.

###### Collection and natural history.

In contrast to the careful capture of the *Lycodon* in Same, our specimen of *Lycodon subcinctus* was obtained within minutes of having been hacked to death at an elementary school. The animal had reportedly been found in the school and was disposed of just as we explained our purpose to some of the local residents. The snake was handed to us draped dead over a branch, with body segments merely attached by threads of skin.

#### Family Homalopsidae – Oriental and Australasian Mudsnakes

##### Cerberus cf. rynchops

(Schneider, 1799)

http://species-id.net/wiki/Cerberus_rynchops

Fig. 25

###### Common names.

(E) Bockadam, Dog-faced Watersnake. *(T) Samea natar (samea = snake, natar = rice paddy).

###### Identification.

**Figure 25.**
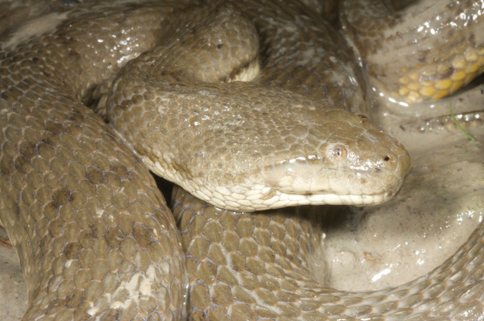
*Cerberus cf. rynchops*. Male (USNM 573675, SVL 598 mm, TL 756 mm) from a rice paddy in the Baucau area, Baucau District. Photo by Mark O’Shea.

Dog-faced watersnakes are easily distinguished based on both morphology and habits. Compared with other snakes, the head is blunt with a rounded snout and relatively small eyes in a dorsolateral position (Fig. 25). Coloration is brownish gray, often with darker blotches on the back. Relative to most snakes in Timor-Leste, these snakes are heavy-bodied (body diameter is robust as opposed to slender). These snakes are most frequently encountered in habitats with standing or slow-flowing water.

###### Collection and natural history.

We captured a single specimen of a snake very similar to *Cerberus rynchops* at night from a flooded rice paddy near Baucau (Baucau District). The snake submerged when illuminated, but was extracted from the muddy water with ease.

###### Taxonomic comment.

In its overall morphology our specimen clearly resembles *Cerberus rynchops*, but a few specific characteristics of its scalation are intermediate between *Cerberus rynchops* and *Cerberus australis*. Until we are able to ascertain its precise taxonomic status, through more detailed morphological and genetic comparisons, we list this specimen as *Cerberus cf. rynchops*. The genus *Cerberus* is currently undergoing a taxonomic evaluation by John C. Murphy (in litt.).

#### Family Typhlopidae – Blindsnakes

##### Ramphotyphlops braminus

(Daudin, 1803)

http://species-id.net/wiki/Ramphotyphlops_braminus

Fig. 26

###### Common names.

(E) Brahminy Blindsnake, Flowerpot Snake. *(T) Samea matan delek (samea = snake, matan delek = blind).

###### Identification.

**Figure 26.**
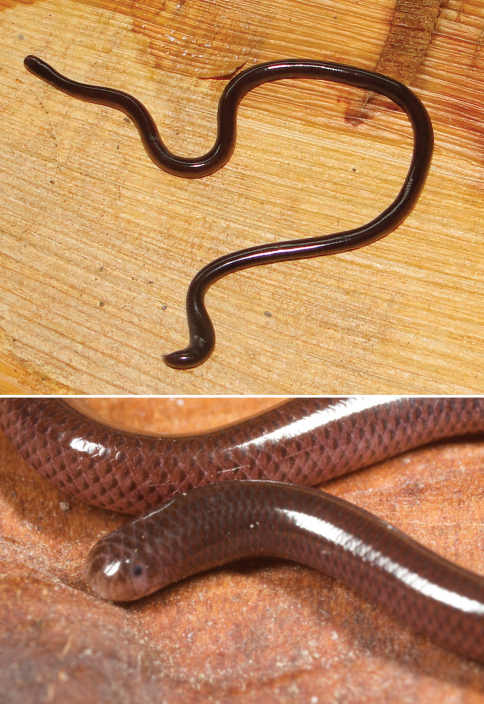
*Ramphotyphlops braminus*. Specimen (USNM 573683, SVL 147 mm, TL 150 mm) from the Same area, Manufahi District. Photos by Hinrich Kaiser (top) and Mark O’Shea (bottom).

Brahminy blindsnakes are vermiform snakes in both morphology (Fig. 26 Upper) and behavior. Body thickness is similar in diameter to the ink tube of a ballpoint pen, and when encountered these snakes will writhe energetically. When grabbed, a typical behavior is to stab the pointed end of the tail into the finger holding the animal in order to gain better purchase for an escape. A closer look will reveal much-reduced eyes as pigmented areas under translucent head scales (Fig. 26 Lower), a tiny forked tongue, and a scale pattern that is diagnostic when differentiating blindsnake taxa. Addison Wynn (USNM) confirmed species identity.

###### Collection and natural history.

We found three specimens of this near-cosmopolitan blindsnake, each in disturbed habitat. The first was found under a rock in the middle of an unpaved country lane with very little vehicular traffic. The second was spotted within minutes of the first under a rock along the edge of the same road. We were surprised by fact that the third specimen essentially found us, by travelling across the smooth, tiled floors of the hotel lobby and into one of our rooms. Even though it was easily spotted, it was quite difficult to pick up.

This is the only known parthenogenetic snake species and this factor, combined with its small size and secretive nature, make it an excellent colonizer. A single specimen arriving in the root-ball of a decorative or food plant is sufficient to establish a new colony. Due to the actions of humans this is the most widely distributed snake species in the world, probably only rivaled by the ubiquitous house geckos (*Hemidactylus* spp.) amongst the lizards.

#### Family Viperidae – True Vipers and Pitvipers

##### Cryptelytrops insularis

(Kramer, 1977)

http://species-id.net/wiki/Cryptelytrops_insularis

Fig. 27

###### Common names.

(E) Lesser Sunda Island Pitviper, Island Pitviper, Lesser Sunda White-lipped Pitviper. (T) Samodok. (Mambae) Samor. (Fataluku) Cuale.

###### Identification.

**Figure 27.**
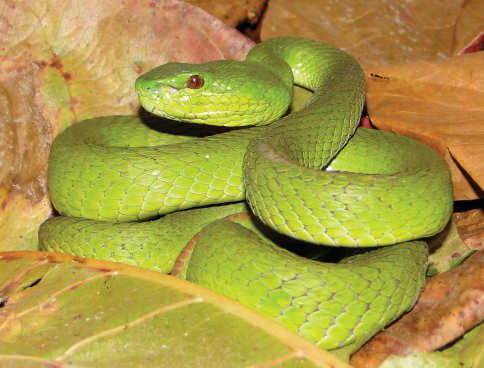
*Cryptelytrops insularis*. Female (USNM [CMD 594], SVL 684 mm, TL 784 mm) from the flood plain of Lake Ira Lalaro, Lautém District. Photo by Hinrich Kaiser.

This snake can be distinguished by both morphology and typical behavior. Characteristic of a pitviper are the paired forward-facing, heat-sensitive pits, posterior to and below to the smaller lateral-facing nares, and the vertically elliptical pupils of the eyes (Fig. 27). Pitvipers documented from Timor-Leste are most frequently bright green in dorsal color with the exception of a reddish stripe on the posterior-most portion of the tail. There exists a second, yellow color morph that apparently is seen in low-rainfall areas in Timor-Leste (CRT, pers. obs.) and also on some of the neighboring islands. Specimens from Wetar are bright yellow whilst some of those from the Komodo Islands are cyan. In the green morph, the labial scales are a yellowish green. The characteristic behavior of these snakes when threatened is to coil the body tightly (Fig. 27) for a defensive strike. These are currently the only venomous reptiles confirmed from Timor-Leste whose bite may have serious implications for humans.

###### Collection and natural history.

We obtained four specimens of this arboreal pitviper in four localities with distinct habitat types, all in eastern Timor-Leste (Baucau, Viqueque, and Lautém Districts). It is remarkable to note that none of the individuals we located were active in trees, shrubs, or leafy vegetation, as might be expected of members of a supposedly arboreal genus, but were located exclusively on the ground. This leads us to speculate that this island form of the widespread green pitviper ecomorph could be less arboreal than some of its congeners and more of a habitat generalist in the absence of competition from terrestrial pitvipers or true vipers, such as the Southeast Asian Russell’s viper (*Daboia siamensis*) that occurs in sympatry with this species further northwest in the Lesser Sundas. The first specimen we collected was a recent road-kill on the Baucau-Lautém road and was found during the daytime within a very short distance of the beach in dry coastal forest. All other specimens were found by night. Our attention was drawn to the second specimen near Timor Village Hotel by a hotel-worker, who encountered it while walking home in an area of short grass adjacent to human habitations. The third specimen (Fig. 27) was encountered while it rested on a bed of decaying foliage in a forested flood plain adjacent to the large open Lake Ira Lalaro flood plain. The last specimen was found at the edge of a dry rice paddy on the outskirts of Baucau. There were no trees within ca. 50 m of this snake’s position. Reports indicate that pitvipers are relatively common in grassy areas and agricultural plots near human habitations, creating a dangerous situation for barefoot humans active during the hours of darkness. The potential snakebite risk posed by this species is being investigated in collaboration with medical colleagues.

###### Taxonomic comment.

This species was regarded as a subspecies of the widespread Asian species *Trimeresurus albolabris* until it was elevated to full species rank by Giannasi et al. (2001). Malhotra and Thorpe (2004) changed the generic assignment from *Trimeresurus* to *Cryptelytrops* in their revision of Asian pitvipers.

### Turtles (Order Testudines)

Our treatment of the turtles of Timor-Leste is primarily concerned with terrestrial species and those living in land-associated aquatic ecosystems. However, six of the world’s seven sea turtle species occur in the northern coastal waters of Australia (*Caretta caretta*, *Chelonia mydas*, *Dermochelys coriacea*, *Eretmochelys imbricata*, *Lepidochelys olivacea*, *Natator depressus*) and these will be recorded as the opportunity arises. We serendipitously observed and filmed a sea turtle, most likely an Olive Ridley (*Lepidochelys olivacea*), in the surf of the Timor Sea off the beach at Loré 1 village (Lautém District). Bleeker (1860) reported specimens of *Chelonia mydas* and *Eretmochelys imbricata* from Wetar Strait.

#### Family Chelidae – South American and Australasian Side-necked Turtles

##### Chelodina timorensis

McCord et al., 2007

http://species-id.net/wiki/Chelodina_timorensis

Fig. 28

###### Common names.

(E) Timor Snake-necked Turtle. *(T) Lenuk kakorok ular (lenuk = turtle, kakorok = neck, ular = snake).

###### Identification.

**Figure 28.**
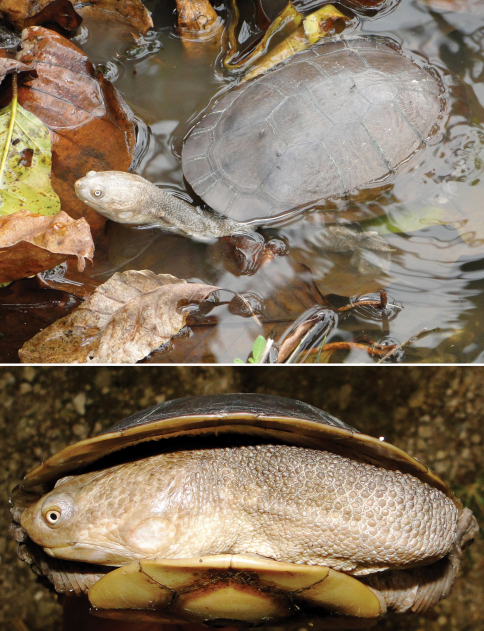
*Chelodina mccordi timorensis*. This specimen was presented to us by a resident of Malahara village, Lautém District. The lower panel shows how this turtle can bend its neck under its carapace when threatened. Photos by Hinrich Kaiser.

Snake-necked turtles are easily distinguished by their long serpentine necks (Fig. 28 Lower), which are fully as long as the entire carapace and which permit the turtle to reach anywhere on its body.

###### Collection and natural history.

During our survey we had heard that local villagers occasionally keep live specimens of this protected snake-necked turtle as status symbols or for trade, a clear contravention of the CITES protocols and an important reason why the government of Timor-Leste is considering acceding to the CITES treaty. The turtle has a highly restricted distribution in Lake Ira Lalaro near the easternmost point of Timor-Leste. The lake itself is primarily seasonal, with water exiting the lake through the Irasequiro River. The river itself does not reach the ocean but disappears beneath an extensive limestone karst escarpment, the Paitxau Range (max. elevation at Mt. Paitxau, 925 m). During our visit we inquired about the availability of a snake-necked turtle for photography, and we learned about a turtle that could be photographed. We declined to purchase the turtle but offered US$ 5 for being given the opportunity to take photographs, in a symbolic gesture and specifically to prove that a living turtle could realize revenue without being traded. This population is closely related to the Roti Island Snake-necked Turtle (*Chelodina mccordi*; Kuchling et al. 2007; McCord et al. 2007).

###### Taxonomic comment.

Even though McCord et al. (2007) described the Lake Ira Lalaro snake-necked turtle population as a distinct species, this decision was not without controversy (see Kuchling et al. 2007), and the new species was not recognized as an acceptable name by the CITES committee. However, the population is still protected as an endangered species under CITES Appendix II. Kuchling et al. (2007) described this population as *Chelodina mccordi timorlestensis*.

#### Family Geoemydidae – South American and Asian Pond Turtles

##### Mauremys reevesii

(Gray, 1831)

http://species-id.net/wiki/Mauremys_reevesii

Fig. 29

###### Common names.

(E) Reeves’ Stripe-necked Turtle, Chinese Pond Turtle. *(T) Lenuk kakorok riskadu (lenuk = turtle, riskadu = striped, kakorok = neck).

###### Identification.

**Figure 29.**
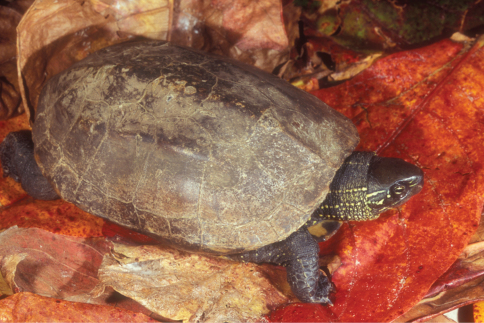
*Mauremys reevesii*. Male individual of the introduced Chinese pond turtle from the Albergaria Planalto in New Town Baucau, Baucau District. Photo by Mark O’Shea.

Chinese pond turtles are readily identified by the characteristic yellow striping and blotching on their necks (Fig. 29).

###### Collection and natural history.

The staff at the Albergaria Planalto, New Town Baucau, became aware of our purpose in collecting specimens of amphibians and reptiles and showed us an unidentified turtle that was kept in a small, stone-encased pond on the grounds of the property. We were told that there were three turtles like this in the area, one collected near Dili, and the two others just across the street in an empty lot at the edge of town. Two of these had escaped by the time of our arrival, but we were able to obtain this specimen as a voucher. The presence of *Mauremys reevesii* was briefly mentioned by McCord et al. (2007), and we reported details regarding its presence in Timor-Leste elsewhere (Kaiser et al. 2010).

### Crocodiles (Order Crocodilia)

#### Family Crocodylidae – Crocodiles

##### Crocodylus porosus

Schneider, 1801

http://species-id.net/wiki/Crocodylus_porosus

Fig. 30

###### Common names.

(E) Saltwater Crocodile, Indo-Pacific Crocodile, Estuarine Crocodile, Naked-necked Crocodile. (T) Lafa’ek tasi (lafa’ek = large lizard, tasi = ocean).

###### Identification.

As the only crocodilian known from Timor and as a creature at the root of the Timorese creation myth, this species probably requires no detailed description. However, *Crocodylus porosus* differs from other crocodiles in the possession of less dermal armor, including lacking the typically four crocodilian post-occipital scutes (between the rear of the skull and a cluster of six nuchal scutes over the shoulders) so obvious on the necks of other species, hence the name ‘naked-necked crocodile’.

###### Collection and natural history.

We saw and photographed several individuals of *Crocodylus porosus* in the wild, most frequently in rivers while driving across bridges. We also saw two captive individuals in Aileu, Aileu District. There exists a substantial population of *Crocodylus porosus* in Lake Ira Lalaro, comprising several hundred individuals with sizes exceeding 3 m in total length (CRT, pers. obs.). Crocodiles are commonly reported from the swamps and swamp forests along the south coast of Timor-Leste, which is the area with the greatest frequency of reported crocodile attacks. Our limited observations on the status of crocodiles in Timor-Leste have been published elsewhere (Kaiser et al. 2009).

**Figure 30.**
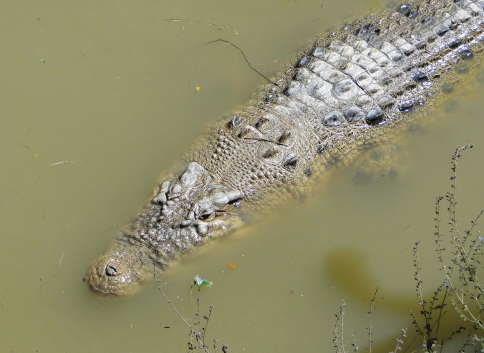
*Crocodylus porosus*. Captive specimen in an enclosure in Aileu, Aileu District. Photo by Hinrich Kaiser.

## Discussion

The emergence of Timor-Leste as an independent nation comes at a precarious time for its natural environment. During the centuries of colonialism and during over two decades of Indonesian occupation, a neglect of environmental management has led to the unsustainable exploitation of natural resources for short-term gain and to the concomitant indiscriminate destruction of habitats. This is perhaps nowhere more strikingly seen than in the disappearance of the famed white sandalwood (*Santalum album*) forests throughout most of Timor-Leste (McWilliam 2005). Even though the new country is including environmental policy as a priority of nation-building, the damage sustained by the infrastructure in the aftermath of the 1999 independence referendum, the lack of capacity to educate about environmental issues, and the near lack of economic activity in many areas of the country place a heavy burden on an already depleted environment (see Sandlund et al. 2001 for a review). Furthermore, the factors traditionally listed when outlining pressures on the environment in tropical developing countries (natural or human-caused habitat loss, soaring population growth, economic need; Bradshaw et al. 2009 and references therein) all apply to newly pacified Timor-Leste.

Perhaps against any expectations of undocumented diversity, our relatively limited survey already shows a level of biodiversity for Timor-Leste that is higher than reported previously and includes considerable endemicity. The occurrence of a species of *Cyrtodactylus* extends the range of this genus to its southeastern extreme. Skinks of the genus *Carlia* appear in a morphological array that constitutes several new species, akin to some *Carlia* “hotspots” in Australia and New Guinea (e.g., Couper et al. 2005) with their substantial cryptic diversity. Wedge skinks (genus *Sphenomorphus*) are a diverse group throughout Southeast Asia, but their high diversity on a single island is noteworthy. Lastly, the diversity of night skinks (genus *Eremiascincus*) appears to have been underestimated on Timor and elsewhere (e.g., Mecke et al. 2009).

Timor-Leste is not unique in this juxtaposition of extensive environmental degradation and a substantial number of undescribed species. This is certainly a Southeast Asian theme (e.g., Giam et al. 2010) but it can be observed in most tropical environments, but especially on some of the nearby islands of Wallacea. Just as in other countries, in which high biodiversity has been discovered despite severe habitat degradation (e.g., islands of the Caribbean: Smith et al. 2005), Timor-Leste poses a conundrum: while biodiversity is high, it is in peril. It is fortunate that the Government of Timor-Leste has established agencies that promote environmental education, the establishment of natural parks and protected zones, and the establishment of sustainable use practices in rural development, and we are hopeful that the threat to the remaining pristine habitats and to the country’s biodiversity can be addressed and managed.

With a view to the realities of human-environment interactions, it is perhaps noteworthy that many of the new species we list appear to possess sufficient phenotypic plasticity to survive or even thrive in degraded habitats. For example, our observations of rice paddy frogs include towns, active and fallow rice paddies, and forested areas. At least one form of night skinks, wedge skinks, and four-fingered skinks occur in, or in close proximity to, villages and their subsistence agricultural plots. The only new species apparently limited to a more pristine or secluded environment was the bent-toed gecko, but even it was collected in a transition zone, between a riverbed and a coffee plantation.

In the habitats we surveyed (e.g., Fig. 2), we were sometimes able to observe a very precise division of ecological niches. For example, in the dry coastal forest in Loré, Lautém District, six different species of lizards (four skinks, the flying lizard, and the monitor lizard) were seen in syntopy. The monitor lizard was seen foraging in the leaf litter, among the groundcover, and also on tree trunks, and among these six species it appears to be the most generalized in its foraging habits. Careful observation of the five other species quickly showed that microhabitat partition existed. Whereas *Carlia* would primarily forage among the dead, dry leaves, in a much more restricted fashion than *Varanus timorensis*, *Sphenomorphus* would occupy the bases, exposed roots, and buttresses of trees. Individuals of *Cryptoblepharus* were usually seen at intermediate heights on the trunks of trees, from 1–4 m high. Some individuals were also seen on thinner branches leading away from the main trunk at comparable heights above the ground. Specimens of *Lamprolepis* occupied the highest position among skinks, usually very close to the transition between the bare trunk and the tree’s foliage or crown. Above the skinks we observed displaying males of *Draco* whose capacity to glide makes this highest position the most desirable.

The results of this survey have been startling as an academic pursuit but also as an historical and cultural journey. The lingering effects of a valiant struggle for independence can temper the excitement of discovering new species, as some of the specimens we collected may have shared their habitat with freedom fighters and their local supporters less than a decade earlier. The interest of both the government of Timor-Leste and the country’s culturally diverse population in supporting those who seek to learn about these habitats has been singularly rewarding and is a positive sign for sustainability and conservation efforts.

## Supplementary Material

XML Treatment for Duttaphrynus melanostictus

XML Treatment for Fejervarya

XML Treatment for Limnonectes timorensis

XML Treatment for Litoria everetti

XML Treatment for Polypedates cf. leucomystax

XML Treatment for Draco timoriensis

XML Treatment for Cyrtodactylus

XML Treatment for Gehyra cf. mutilata

XML Treatment for Gekko gecko

XML Treatment for Hemidactylus frenatus

XML Treatment for Hemidactylus platyurus

XML Treatment for Carlia

XML Treatment for Cryptoblepharus leschenault

XML Treatment for Eremiascincus

XML Treatment for Eutropis cf. multifasciata

XML Treatment for Lamprolepis cf. smaragdina

XML Treatment for Sphenomorphus

XML Treatment for Varanus timorensis

XML Treatment for Coelognathus subradiatus

XML Treatment for Dendrelaphis inornatus timorensis

XML Treatment for Lycodon capucinus

XML Treatment for Lycodon subcinctus

XML Treatment for Cerberus cf. rynchops

XML Treatment for Ramphotyphlops braminus

XML Treatment for Cryptelytrops insularis

XML Treatment for Chelodina timorensis

XML Treatment for Mauremys reevesii

XML Treatment for Crocodylus porosus

## Appendix I. Specimens Examined

In the following enumeration, specimens are listed using the collection acronym and a specimen number. The specimen number is composed either of numbers only, or of capital letters followed by numbers. The letters indicate the field numbers of Stephen J. Richards (SJR) or Christine M. Kaiser (CMD). Specimens with field numbers have not been accessioned yet at their respective depositories, but the specimens will nevertheless be accessible via a database search of field numbers at these institutions.

### Frogs and Toads

**Family Bufonidae**

*Duttaphrynus melanostictus*.—SAMA [SJR 7127], USNM 537650–53

**Family Dicroglossidae**

*Fejervarya* sp. 1 [Ca CMD 431].—USNM [CMD 429, 431, 435–36, 508, 510, 516–17, 538, 540–41, 543–45].

*Fejervarya* sp. 2 [Ca CMD 507].—USNM [CMD 410, 426, 433–34, 506–07, 512–15, 519, 533–36, 539, 542].

*Fejervarya* sp. 3 [Ca CMD 355] .—USNM [CMD 355, 382, 392, 428, 430, 432, 469–70, 509, 511, 518, 568].

*Limnonectes timorensis*.—SAMA [SJR 7072, 7075–78, 7092–93, 7099–100, 7108–10, 7112–13, 7117, 7122, 7128–30, 7134–39], USNM [CMD 422–23].

**Family Hylidae**

*Litoria everetti*.—USNM [CMD 420–21].

**Family Rhacophoridae**

*Polypedates cf. leucomystax*.—SAMA [SJR 7073–74, 7079–82, 7102–06, 7118–21, 7131–33, 7140], USNM [CMD 357–59, 381, 419, 427, 457, 464–67, 497–502].

### Lizards

**Family Agamidae**

*Draco timoriensis*.—USNM 573658–63.

**Family Gekkonidae**

*Cyrtodactylus* sp. 1 [Ca CMD 383].—USNM [CMD 383–84].

*Gehyra cf. mutilata*.—SAMA [SJR 7070–71], USNM [CMD 459–60, 505].

*Gekko gecko*.—SAMA [SJR 7094–95], USNM 573671–74.

*Hemidactylus frenatus*.—USNM [CMD 441, 476–78, 488–90, 524–26].

*Hemidactylus platyurus*.—USNM [CMD 437–38, 458, 494, 503–04].

**Family Scincidae**

*Carlia* sp. 1 [Ca CMD 354].—USNM [CMD 354, 360–63, 368–69, 371, 403, 406, 412–13].

*Carlia* sp. 2 [Ca CMD 400].—USNM [CMD 400–02, 404–05, 407–08, 411, 414].

*Carlia* sp. 3 [Ca CMD 471].—USNM [CMD 471–73, 483–85].

*Carlia* sp. 4 [Ca CMD 522].—USNM [CMD 522].

*Carlia* incertae sedis.—SAMA [SJR 7001–04, 7068–69, 7085–86, 7098, 7116, 7123–25.

*Cryptoblepharus leschenault*.—USNM 573654–57.

*Cryptoblepharus* incertae sedis.—SAMA [SJR 7083–84

*Eremiascincus* sp. 1 [Ca CMD 365].—USNM [CMD 365–67, 370, 372–74, 385–88, 393–99].

*Eremiascincus* sp. 2 [Ca CMD 474].—USNM [CMD 474, 486–87].

*Eremiascincus* incertae sedis.—SAMA [SJR 7056–67, 7097, 7107, 7114–15, 7126].

*Eutropis cf. multifasciata*.—SAMA [SJR 7088–91, 7096], USNM 573664–666.

*Lamprolepis cf. smaragdina*.—USNM 573667–70.

*Sphenomorphus* sp. 1 [Ca CMD 445].—USNM [CMD 445–49, 451–55].

*Sphenomorphus* sp. 2 [Ca CMD 356].—USNM [CMD 356, 364].

*Sphenomorphus* sp. 3 [Ca CMD 415].—USNM [CMD 415].

*Sphenomorphus* sp. 4 [Ca CMD 416].—USNM [CMD 416].

*Sphenomorphus* incertae sedis.—SAMA [SJR 7111].

### Snakes

**Family Colubridae**

*Coelognathus subradiatus*.—USNM 573676.

*Dendrelaphis inornatus timorensis*.—USNM [CMD 450, 468, 493].

*Lycodon capucinus*.—SAMA [SJR 7087], USNM 573681.

*Lycodon subcinctus*.—USNM 573682.

**Family Homalopsidae**

*Cerberus rynchops*.—USNM 573675.

**Family Typhlopidae**

*Ramphotyphlops braminus*.—USNM 573683–85.

**Family Viperidae**

*Cryptelytrops insularis*.—USNM 573677–680.

### Turtles

**Family Geoemydidae**

*Mauremys reevesii*.—USNM 573649.

## Appendix II. Glossary

Glossary of relevant scientific terms. (doi: 10.3897/zookeys.109.1439.app) File format: Microsoft Word (doc).

**Explanation note:** A glossary of relevant scientific terms is provided to readers unfamiliar with some of the terms used in our report.

Copyright notice: This dataset is made available under the Open Database License (http://opendatacommons.org/licenses/odbl/1.0/). The Open Database License (ODbL) is a license agreement intended to allow users to freely share, modify, and use this Dataset while maintaining this same freedom for others, provided that the original source and author(s) are credited.
